# How people evaluate anti-corona measures II—Lessons for future crises from a longitudinal study and qualitative analysis

**DOI:** 10.3389/fpsyg.2026.1806942

**Published:** 2026-08-28

**Authors:** Hendrik Godbersen, Susana Ruiz-Fernández

**Affiliations:** 1 FOM Hochschule für Oekonomie and Management, Essen, Germany; 2 Department of Psychology, Brandenburg University of Technology, Cottbus-Senftenberg, Germany

**Keywords:** COVID-19 mitigation measures, COVID-19 pandemic, COVID-19 vaccination, crisis management, evaluation of anti-corona measures, longitudinal study and qualitative analysis of perception of anti-Corona measures, Means-End Theory of Complex Cognitive Structures, Theory of Planned Behavior

## Abstract

The COVID-19 pandemic represents an unprecedented health crisis in modern times. The pandemic itself and governmental counter-measures severely affected the individual and social lives of people around the globe. Against this backdrop, we examined how people evaluated the main anti-corona measures — restrictions on outdoor activities, tips for hygiene, and tips for mental health — with regard to the elements of the Theory of Planned Behavior (attitude, subjective norm, and perceived behavioral control) and four social spheres (close family, wider family and friends, colleagues at work, and society in general). The main objective of this research was to establish how these evaluations changed over the course of the pandemic. Additionally, we examined the evaluation of vaccinations introduced at a later stage and aimed to identify the subjective reasons for people's evaluations of these measures. Accordingly, we compared evaluations of early anti-corona measures at the beginning of the pandemic (March–April 2020; *n* = 663) with evaluations roughly 1 year later (January–March 2021; *n* = 577). Vaccination was examined in the second measurement period. The methodological basis of our measurements is the Means-End Theory of Complex Cognitive Structures, through which we determined the perceived relevance and quality of the measures with regard to the social spheres and elements of the Theory of Planned Behavior. Furthermore, we qualitatively examined subjective reasons for positive and negative evaluations through open questions. Results reveal that people's expectations (subjective relevance) did not substantially change over time. Protection from the coronavirus and its consequences (attitude) was regarded as more important than practicability (perceived behavioral control), which in turn outweighed willingness to fulfill others' expectations (subjective norm). People attributed higher relevance to their close families than to other social spheres. Regarding perceived quality, hygiene tips received the highest ratings, followed by restrictions on outdoor activities and vaccination, while mental health tips were rated lowest. All quality perceptions declined during the pandemic. Overall, results suggest that crisis measures should have clear objectives with substantial benefits, practicability in daily life, a basis in scientific evidence, and clear explanation. In summary, people prefer simple (though not necessarily easy) solutions to severe crises.

## 1 Introduction

We published a study in 2020 (Godbersen et al., 2020), which examined how people evaluate the main anti-Corona measures during the early stages of the COVID-19 pandemic. In our current study, we use the data of our 2020 study and new data we collected in 2021 to investigate how the people's evaluation of the early anti-Corona measures, i.e., restrictions on outdoor activities, tips for hygiene and tips for mental health, changed over the course of the COVID-19 pandemic. Additionally, we examine the people's evaluation of vaccinations, which were only available by the end of 2020. Furthermore, we qualitatively examine the subjective reasons for the people's evaluation of the main anti-Corona measures. On this basis, we aim to give advice on how to deal with future crises from the people's perspective. The theoretical basis for our research is laid in this section. We briefly describe the COVID-19 pandemic and explain the main anti-Corona measures in Section 1.1. In Section 1.2, we lay the foundation for our hypothesized model, which, alongside our research questions, is presented in Section 1.3.

### 1.1 The COVID-19 pandemic and (governmental) anti-Corona measures

We describe the COVID-19 pandemic, the early anti-Corona measures and the vaccination in the following subsections.

#### 1.1.1 The COVID-19 pandemic

The World Health Organization WHO (2020) declared the outbreak and spread of the severe acute respiratory syndrome 2 (SARS-CoV-2) a pandemic in March 2020. The pandemic spread rapidly and led to quickly rising infections in virtually every country of the globe (Phan, 2020; Sohrabi et al., 2020; Velavan and Meyer, 2020).

The severity of this health crisis becomes apparent when looking at the substantially increased mortality during the COVID-19 pandemic. Amongst others, Msemburi et al. (2023) estimate 14.8 million excess deaths for the years 2020 and 2021, whilst Wang et al. (2022) estimate 18.2 million excess deaths for the same time period. Another indicator for the negative impact the COVID-19 pandemic had on physiological health of people is the prevalence of Long COVID, which researchers and politicians were not aware of at the start of the pandemic. In this context, Woodrow et al. (2023) estimate that 34.5% did not return to their full health and fitness level 12 weeks after their infection with COVID-19.

The COVID-19 pandemic did not only affect the physical health. The subjectively perceived threat of the pandemic and the measures to slow or contain the spread of the coronavirus also negatively impacted the mental health with an increase of depressions, anxieties, sleep disorders, posttraumatic stress disorders, burnouts, psychological distress and suicidal ideations, amongst others (Kauhanen et al., 2023; Kunzler et al., 2023; Kupcova et al., 2023; Peng et al., 2023; Planchuelo-Gómez et al., 2020; Salimi et al., 2023; Tong et al., 2023).

#### 1.1.2 Early anti-Corona measures

Against the backdrop of an in modern times unprecedented global health crisis, virtually every country around the globe took measures to mitigate the spread of the COVID-19 pandemic. As the coronavirus is primarily transmitted through direct personal contact and respiratory droplets (Guerrero et al., 2020; Rothan and Byrareddy, 2020; Wang and Du, 2020) and no vaccine or therapeutic medicine was developed at the start of the pandemic (Ahmed et al., 2020; Liu et al., 2020; Lurie et al., 2020; Rothan and Byrareddy, 2020), many countries introduced social or spatial distancing measures, i.e., decreasing social contacts and increasing the space between people (Abel and McQueen, 2020; Kissler et al., 2020; Lewnard and Lo, 2020; Sen-Crowe et al., 2020). The aim of such restrictions on outdoor activities is to slow the spread of the virus so that the healthcare system is still capable of treating a comparably lower number of infected patients (Balasa, 2020; Sen-Crowe et al., 2020). Some countries, like Sweden, introduced restrictions on outdoor activities mainly in the form of recommendations (Juranek and Zoutman, 2020), whilst other countries, like Italy, enforced restrictions on outdoor activities through lockdowns (Sjödin et al., 2020).

In Germany, the federal government and the governments of the German states introduced several social or spatial distancing measures during the first phases of the COVID-19 pandemic. On 23 March 2020, a lockdown was introduced (Merkel, 2020a), which, at its core, prescribed a spatial distance to others of at least 1.5 m, allowed only to meet one person outside one's own household, and ordered the closure of restaurants, cafés and other service providers. On 06 May 2020, the federal government and the government of the German states decided to gradually lift the first lockdown and to regionally react to the development of the pandemic, depending on regional case numbers (Merkel, 2020b). On 28 October, the federal government and the governments of the German states decided to re-introduce, amongst other measures, restrictions on meeting persons from other households and the closure of restaurants, cafés, other service providers and entertainment events (Merkel, 2020c). These measures were tightened on 16 December 2020 (Merkel, 2020d), e.g., tighter restrictions on social contacts and closure of non-food retailers. On 03 March 2021, the federal government and the government of the German states decided to gradually lift the second lockdown and to regionally react to the development of the pandemic, depending on regional case numbers**—**similar to the decision made roughly a year before (Merkel, 2021).

Even though social or spatial distancing measures were arguably at the core of the early anti-Corona strategies, complementing measures, such as hygiene measures, were also introduced to slow the spread of the coronavirus (e.g., Bruinen de Bruin et al., 2020; Sen-Crowe et al., 2020). In Germany, the Federal Center for Health Education recommended to use a paper tissue or the crock of the arm when sneezing or coughing, to keep hands away from the face, to stay away from people who have a cough, cold or fever, to avoid touching other people, and to regularly and thoroughly wash hands (BzgA, 2020).

The threat of the COVID-19 pandemic itself and the respective mitigation measures can negatively impact mental health and mental wellbeing, as mentioned in Section 1.1.1. The negative psychological effects of uncertain situations, a perceived mass threat and feelings of isolation on mental health could be documented even before the COVID-19 pandemic, (e.g., Dar et al., 2017; Mak et al., 2009; Zhou et al., 2019). Against this backdrop, Mental Health Europe (2020) introduced tips for mental health, which were also available as a German version. It was recommended to seek accurate information about the COVID-19 pandemic from legitimate sources, to set limits for the reception of news about COVID-19, to look after oneself through good hygiene, healthy eating, enough sleep, daily routines for mental health and enjoyable activities, to reach out to others for support, to maintain a sense of hope and positive thinking, to acknowledge one's feelings and allow feelings of stress, anxiety or depression, to talk to one's children, and to ask for professional support if required.

#### 1.1.3 Vaccination

We pointed out in the previous section that no vaccine or therapeutic medicine was available at the start of the COVID-19 pandemic. However, the development process of a COVID-19 vaccine was sped up in 2020 (Ndwandwe and Wiysonge, 2021; Rabaan et al., 2020; Uttarilli et al., 2021). By the end of 2020 and the start of 2021, several vaccines were globally available and administered. In Germany the vaccination campaign started by the end of December 2020 (Bundesregierung, 2021). To date (12 July 2024), 5.47 billion doses were administered globally (WHO, 2024) and 197 million doses in Germany (Robert Koch Institute, 2024). These numbers indicate that the COVID-19 vaccination was introduced as the main measure to combat the pandemic, especially in developed countries such as Germany. However, not the entire population shares the perception of the vaccination as a “panacea” for the COVID-19 pandemic, as the vaccination ratio of 76.4% in Germany as of 08 April 2023 indicates (Bundesministerium für Gesundheit, 2023).

Several studies reveal concerns about the safety, negative side effects, rapid development, lack of enough clinical trials and effectiveness of vaccine as the main determinants of vaccine hesitancy (e.g., Adu et al., 2023; Fares et al., 2021; Kafadar et al., 2023; Koskan et al., 2023). On the other hand, the trust in the COVID-19 vaccine development and approval process, the perceived benefits and effectiveness of the COVID-19 vaccine, its perceived safety, and the perceived severity and risk of the coronavirus positively influence the intention to get vaccinated (e.g., Burke et al., 2021; Coe et al., 2022; Fares et al., 2021; Koskan et al., 2023).

### 1.2 Subjective evaluation of anti-Corona measures

We lay the theoretical basis for our hypothesized model on how people evaluate the afore-described anti-Corona measures in the following two subsection. Firstly, we elaborate on the social spheres that are affected by the COVID-19 pandemic and the respective counter measures. Secondly, we introduce the Theory of Planned Behavior (Ajzen, 1985, 1988, 1991), which will structure the evaluation model. A more detailed development of our hypothesized model can be found in Godbersen et al. (2020), to which this study is a follow-up.

#### 1.2.1 Subjective evaluation of anti-Corona measures within the social spheres

A social component is inherent in a pandemic, as the virus is transmitted through contacts between people (Guerrero et al., 2020; Rothan and Byrareddy, 2020; Wang and Du, 2020). At the same time, the anti-Corona measures of the early phase of the pandemic, especially restrictions on outdoor activities, have an impact on social lives by reducing social contacts, as described in Section 1.1.2. Alizadeh et al. (2023) show that the COVID-19 pandemic and, with it, the respective mitigation measures negatively impacted social capital and social relationships and increased social vulnerability. Similarly, the COVID-19 pandemic and social or spatial distancing, especially lockdowns, fostered psychological distress (King et al., 2023) and social anxiety (Kindred and Bates, 2023). On the other hand, social connectedness promoted individual wellbeing during the COVID-19 pandemic (Brown and Leite, 2023).

The social dimension of health is not limited to the COVID-19 pandemic or any other infectious disease. It is rather plausible to understand health in general not only as a biomedical model but also as being embedded in social contexts (Germov, 2014), as the individual lifestyle within a social setting, and within living, working and general socioeconomic conditions affects the health of an individual et vice versa (Dahlgren and Whitehead, 1991). Against this backdrop and in line with our first study (Godbersen et al., 2020), we propose four social spheres being relevant for the people's perception of the COVID-19 pandemic and their evaluation of anti-Corona measures:

- Close family.
- Wider family and friends.
- Colleagues at work.
- Society in general.

According to the categorization of groups by Lickel et al. (2000), the close family, and the wider family and friends represent the intimacy groups, the colleagues at work represent a task group, and the society in general represents a social category or loose association.

#### 1.2.2 Subjective evaluation of anti-Corona measures within the theory of planned behavior

In the previous sections, we pointed out that the perception and effects of the COVID-19 pandemic as well as the evaluation and adherence to anti-Corona measures is embedded in both, individual cognitive and behavioral processes as well as social contexts. In line with our first study (Godbersen et al., 2020), we propose the elements of the Theory of Planned Behavior (Ajzen, 1985, 1988, 1991) as the basic structure for the subjective evaluation of anti-Corona measures because its elements entail individual evaluations of attitudinal objects, perceived social influences and integrations of behaviors in one's (social) life.

The original Theory of Planned Behavior (Ajzen, 1985, 1988, 1991) aims at predicting behavioral intentions but can be adjusted to explaining the evaluation of virtually any attitudinal object, as principally related theories show, e.g., motivational theories (Atkinson, 1964), attitudinal theories (Fishbein, 1963) and the Means-End Theory of Complex Cognitive Structures (Godbersen, 2016, 2019; Godbersen and Barluschke, 2020; Godbersen and Kaupp, 2019; Godbersen and Wenzel, 2022), which will be applied in our research. Thus, the Theory of Planned Behavior (Ajzen, 1985, 1988, 1991) is integrated in this study as a structuring framework that is directed toward the evaluation of the anti-Corona measures and not as a predictor of behavioral intentions, contrary to its original conception.

The elements of the Theory of Planned Behavior (Ajzen, 1985, 1988, 1991) and their definition in the context of this study are:

- Attitude is the positive or negative evaluation of an attitudinal object. In the context of an anti-Corona measure, attitude can be understood as the perceived protection from the coronavirus and its consequences.
- Subjective norm is the perceived social pressure with regard to an attitudinal object. In the context of an anti-Corona measure, subjective norm can be understood as the willingness to fulfill the expectations of others.
- Perceived behavioral control is the perceived capacities and constraints of an individual with regard to an attitudinal object. In the context of an anti-Corona measure, perceived behavioral control can be understood as the practicability of the anti-Corona measure.

The application of the Theory of Planned Behavior in the evaluation of anti-Corona measures is supported by its predictive value regarding health-conscious behavior and decisions in general (e.g., Blue, 1995; Godin and Kok, 1996; Lajunen and Räsänen, 2004; O'Connor and Armitage, 2003; Povey et al., 2000; Simşekoglu and Lajunen, 2008). More particular with regard to anti-Corona measures, the Theory of Planned Behavior (Ajzen, 1985, 1988, 1991) could explain the intentions to vaccinate (Bui et al., 2023; Wolff, 2021; Xie et al., 2023) and the intentions to follow social or spatial distancing (e.g., Frounfelker et al., 2021; Gibson et al., 2021; Wollast et al., 2021), amongst others.

### 1.3 Hypothesized model and research questions

We showed in Section 1.1 that the restrictions on outdoor activities, tips for hygiene, tips for mental health and vaccination were among the main measures to counter the COVID-19 pandemic globally and in Germany in particular. Thus, we chose to examine how people evaluate these anti-Corona Measures.

In Section 1.2, we pointed out that the afore-mentioned anti-Corona measures can affect the social spheres of people, namely the close family, wider family and friends, colleagues at work, and the society in general. Furthermore, we introduced the Theory of Planned Behavior (Ajzen, 1985, 1988, 1991) to structure the people's assessment of anti-Corona measures.

**Figure 1:**
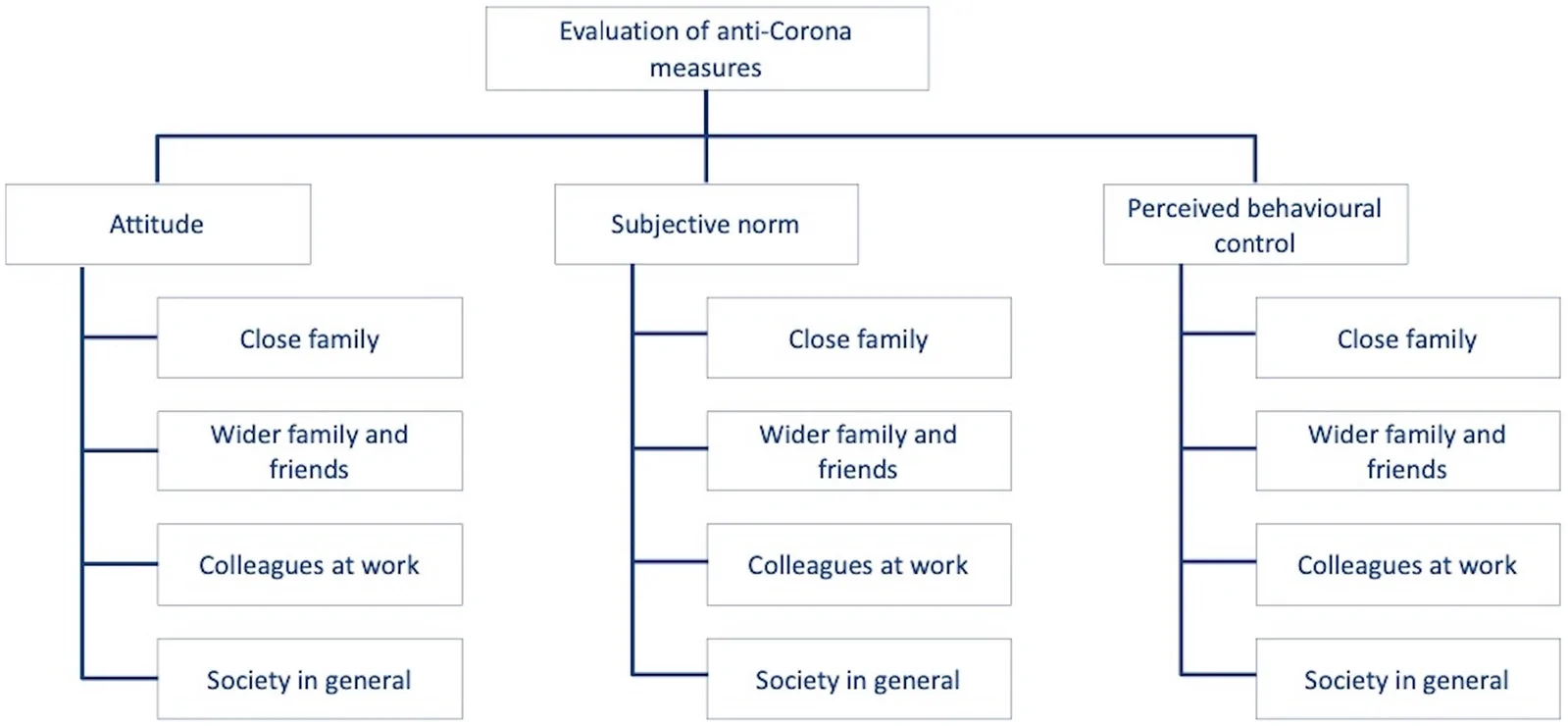
Model of people's evaluation of anti-Corona measures (Godbersen et al., 2020).

Against this backdrop, we developed and validated a model representing the people's evaluation of anti-Corona measures that consists of three levels in a previous study (Godbersen et al., 2020; see Figure 1). The overall evaluation of an anti-Corona measure is situated on the top level. The second level of our model consists of the elements of the Theory of Planned Behavior (Ajzen, 1985, 1988, 1991): attitude (which can be understood as the perceived protection from the coronavirus and its consequences), subjective norm (which can be understood as the willingness to fulfill the expectations of others) and perceived behavioral control (which can be understood as the practicability of anti-Corona measures). On the third and most concrete level of our model, we understand the social spheres—close family, wider family and friends, colleagues at work, and the society in general—as subordinated elements to the afore-mentioned constructs of the Theory of Planned Behavior (Ajzen, 1985, 1988, 1991). Our model implies that the evaluation of an element on a higher level depends on the evaluation of its subordinated elements. Following the expectancy value theories (e.g., Atkinson, 1964; Fishbein and Ajzen, 1975; Vroom, 1995) and the afore-described Theory of Planned Behavior (Ajzen, 1985, 1988, 1991), which can be seen as a specific application of an expectancy value theory, it is not only important how well people evaluate the elements of a model but also how relevant these elements are to them. Thus, we examine the subjective relevance and perceived quality of the elements of our hypothesized model.

We could examine how people evaluate anti-Corona measures during the first phase of the pandemic and introduction of counter-measures in a previous project (Godbersen et al., 2020). However, the COVID-19 pandemic and, with it, the counter-measures were in effect since the beginning of 2020 and, therefore, had an impact on people over a prolonged period of time, as shown in Section 1.1. In the context of other general or personal crises, studies could show that the severity and/or longitude of such crises can have a negative effect on the mental health and wellbeing of people over time. Examples are the financial crisis of 2008 (Lindström and Giordano, 2016; Sargent-Cox et al., 2011) and long-term unemployment (Thern et al., 2017) or subjective financial difficulties (Skapinakis et al., 2006).

This arises the questions if and how the perception and evaluation of the anti-Corona measures have changed during the pandemic. Using the data of our first research in 2020 (Godbersen et al., 2020) and integrating a follow-up study in 2021, we examine such longitudinal effects for the restrictions on outdoor activities, tips for hygiene and tips for mental health. Vaccinations were only available at the end of 2020 so that a longitudinal examination is not possible in this research. Against this backdrop, we formulate the following research questions:

- RQ1 (subjective relevance): How did the relevance of the attitude, subjective norm and perceived behavioral control as well as the relevance of the social spheres develop for the people's evaluation of anti-Corona Measures during the COVID-19 pandemic?
- RQ2.1 (subjective quality of restrictions on outdoor activities, tips for hygiene and tips for mental health): How did the people's evaluation of the anti-Corona measures with regard to their attitude, subjective norm and perceived behavioral control as well as their social spheres develop during the COVID-19 pandemic?
- RQ2.2 (subjective quality of the vaccination): How did the people evaluate the vaccination with regard to their attitude, subjective norm and perceived behavioral control as well as their social spheres?
- RQ3.1 (optimisation of the restrictions on outdoor activities, tips for hygiene and tips for mental health): How did the potential of, the need for and the priority of increasing the effectiveness of anti-Corona Measures with regard to the attitude, subjective norm and perceived behavioral control as well as the social spheres develop from the people's perspective during the COVID-19 pandemic?
- RQ3.2 (optimisation of the vaccination): What was the potential of, the need for and the priority of increasing the effectiveness of the vaccination with regard to the attitude, subjective norm and perceived behavioral control as well as the social spheres from the people's perspective?

In addition to these quantitative research objectives, we aim to determine the subjective reasons for positive and negative evaluations of anti-Corona measures through a qualitative research approach. Thus, we formulate the following complementing research question:

- RQ4 (qualitative analysis of the people's reasons for their evaluation of anti-Corona measures): What were the people's reasons for their positive and negative evaluation of the anti-Corona measures restrictions on outdoor activities, tips for hygiene, tips for mental health and vaccination?

## 2 Material and methods

The research design and the measurement instruments, we applied, are explained in this section.

### 2.1 Research design

We used a standardized online questionnaire in both studies. Study 1, whose results are already published in Godbersen et al. (2020), was conducted from 25 March to 15 April 2020 and Study 2 from 15 January to 07 March 2021; meaning that the first study fell into the first lockdown in Germany, whilst the second study fell into the second lockdown. The participants of both studies were students of FOM University of Applied sciences in Germany. Students at FOM are part-time students who parallelly work in regular jobs. Study 1 resulted in 663 completed questionnaires and Study 2 in 577 completed questionnaires. The average age of the participants of Study 1 is 26.73 years (SD = 5.03), whilst the average age of the participants of Study 2 equals 26.30 years (SD = 4.73). The sample of Study 1 consists of 25.34% males and 74.66% females; the sample of Study 2 consists of 24.44% males, 75.39% females and 0.17% diverse. We conducted a *t*-test and a chi^2^-test to examine potential differences in the sociodemographic structure of our two samples. With a *p*-values of 0.1223 for age and 0.5295 for gender, we could not establish significant differences between Study 1 and Study 2 regarding sociodemographic characteristics.

### 2.2 Measurement

Our hypothesized model, presented in Section 1.3, forms the core content of and basis for our empirical research. To answer the research questions RQ1 to RQ3.2 (subjective relevance, subjective quality and optimisation of anti-Corona measures), the Means-End Theory of Complex Cognitive Structures, which will be explained in the next subsection, is applied. Research question RQ4 (people's reasons for their evaluation of anti-Corona measures) is answered through a qualitative analysis, presented in the second subsection. In the third subsection, we will introduce a few additional variables we examined to gain a broader and more complete understanding of the people's evaluation of anti-Corona measures.

#### 2.2.1 Measurement with the Means-End Theory of Complex Cognitive Structures

We used the same questions in Study 2 as we did in Study 1 to be able to compare the results and possible changes. The participants had to rate the subjective relevance of the model elements (i.e., the elements of the Theory of Planned Behavior and the social spheres) on a continuous rating scale from 0 (not important) to 100 (very important). To determine the subjective quality of the anti-Corona measures, we also used continuous rating scales, ranging from 0 (not good) to 100 (very good). For each of the anti-Corona measures, the participants had to rate how good the respective measure is to protect the close family, wider family and friends, colleagues at work, and society in general (attitude); how well these groups evaluate the respective anti-Corona measure (subjective norm); and how practical the anti-Corona measure is for the participants in these four social spheres (perceived behavioral control). As mentioned in Section 1.3, we examined the restrictions on outdoor activities, tips for hygiene, tips for mental health and vaccination as the main anti-Corona measures in Study 2; in Study 1, however, we only examined the first three anti-Corona measures, as vaccinations were neither developed nor available at this early stage of the COVID-19 pandemic.

Based on these measurements, the analysis was conducted according to the Means-End Theory of Complex Cognitive Structures (Godbersen, 2016, 2019; Godbersen and Barluschke, 2020; Godbersen et al., 2020; Godbersen and Kaupp, 2019) and is described in detail in the publication of our first study (Godbersen et al., 2020). At the core of the Means-End Theory of Complex Cognitive Structures is the following equation:


$$\begin{array}{l} cQ_{i}=\overset{n}{\underset{j=1}{\sum }}{\frac{V_{j}}{\sum_{j=1}^{n}V_{j}}}^{*}eQ_{j} \end{array}$$

*cQ*_*i*_**—**calculated quality of element i on the middle level of the model (attitude, subjective norm and perceived behavioral control)

*eQ*_*j*_**—**empirical quality of element j on the lowest level of the model (close family, wider family and friends, colleagues at work, and society in general within the attitude, subjective norm and perceived behavioral control)

*V*_*j*_**—**perceived value of element j (empirically measured) on the lowest level of the model (close family, wider family and friends, colleagues at work, and society in general within the attitude, subjective norm and perceived behavioral control)

This equation indicates that the subjective qualities for the middle level (attitude, subjective norm and perceived behavioral control) and the highest level (overall evaluation of the respective anti-Corona measure) of our hypothesized model are calculated rather than measured in the questionnaire. In essence, the quality of a model element equates to the sum of the qualities of its subordinated elements, weighed with their standardized subjective relevance (first term of the sum in the afore-presented equation). In plain terms and using the example of the perceived protection of an anti-Corona measure, the equation means that the perceived quality of the anti-Corona measure is calculated as the weighted average of how well the measure works for each social sphere, with the weights reflecting how important each social sphere is to the individual. Within the Means-End Theory of Complex Cognitive Structures, this standardized subjective relevance is called normed value and indicates the effect of an element on its superior element on the next level of the model; thus, the normed values can be compared to regression coefficients. To determine the effect of an element, situated on the lowest level of the model, on the element on the top level, total normed values are calculated by multiplying the normed value of an element on the lowest level of the model with the normed value of its related element on the middle level of the model.

**Figure 2:**
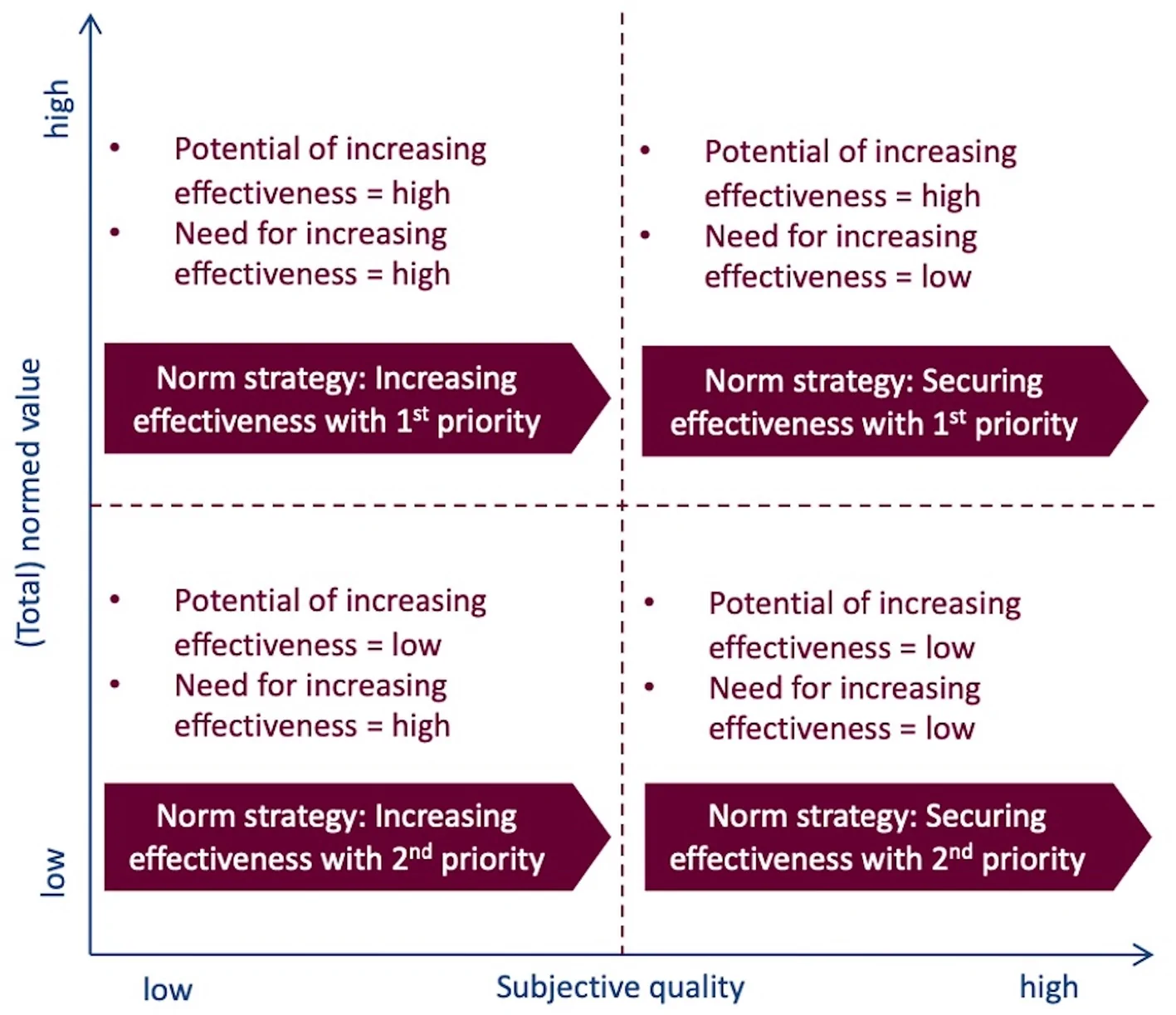
Norm strategies of the Means-End Theory of Complex Cognitive Structures depending on quality and (total) normed value (Godbersen, 2019; Godbersen et al., 2020).

The measured and calculated qualities and the normed and total normed values of the model elements can be combined in a matrix to derive norm strategies for optimizing the examined anti-Corona measures, as shown in Figure 2. The (total) normed values correspond with the potential of increasing the effectiveness of an anti-Corona measure with regard to the respective element, whilst the subjective qualities correspond with the need for increasing the effectiveness of an anti-Corona Measure with regard to the respective element.

As we applied a longitudinal research design with independent but similar samples (please see Section 1.3), we used independent sample *t*-tests to test for significant differences between Study 1 and Study 2. This analysis was not applied with regard to vaccination, as COVID vaccinations were only available during our second study. We conducted our analysis in R (R Development Core Team, 2017).

#### 2.2.2 Qualitative analysis of people's reasons for their evaluation of anti-Corona measures

As stated in research question RQ4, we also aim in this study to find the reasons why people evaluate anti-Corona measures positively or negatively based on their subjective perspective, following in the research tradition of the Grounded Theory (Glaser and Strauss, 2017). To this end, we asked the participants to state their reasons for their positive or negative evaluation of each anti-Corona measure (restrictions on outdoor activities, tips for hygiene, tips for mental health and vaccination) in an open question during Study 2.

With regard to the analysis, we followed the approach of the Guideline of Qualitative Analysis and Model Development by Godbersen (2024), which is based on the Qualitative Content Analysis (Kuckartz and Radiker, 2023; Mayring, 2022) and the Grounded Theory Methodology by Strauss and Corbin (1998), and could be applied in qualitative research projects (Godbersen, 2026; Godbersen and Ruiz Fernández, in press). In the first step we generally familiarized ourselves with the data to gain an overview of the answers. In the second step, we defined three main categories according to our research objectives, i.e., positive evaluation, criticism, and ambiguous or not applicable, and applied them to the data. In the third and main step of the qualitative analysis, we inductively coded the participants' statements, i.e., we developed categories from the answers of our participants, and regularly evaluated if the inductive categories can be summarized into categories on a more abstract level. This inductive coding can be compared to open coding of the Grounded Theory Mehodology by Strauss and Corbin (1998). Additionally, we assessed if the more abstract categories incorporate the more specific ones and if the categories are consistent and distinctive from each other, which resembles axial and selective coding of the Grounded Theory Methodology by Strauss and Corbin (1998). The coding was predominantly conducted bei the first author, with the second author assisting through plausibility checks. The result of our qualitative analysis is a hierarchical system of main categories on a more abstract level and subordinated categories on a more specific level. In the fourth and final step, we calculated the frequencies of the categories' ocurences for the four examined anti-Corona measures as the percentage of the total number of participants.

#### 2.2.3 Further variables

We examined additional variables to supplement the findings with the Means-End Theory of Complex Cognitive Structures and the qualitative analysis. To this end, we ask the participants in Study 1 (Godbersen et al., 2020) and Study 2 how well they feel informed about the COVID-19 pandemic, how important several information sources are for them (close family, wider family and friends, colleagues at work, classic media, and new media), how well they perceive information from the government and from researchers or research institutes, and as how threatening they perceive COVID-19 for their social spheres. For all of these measurements, we applied continuous rating scales from 0 to 100 with pole descriptions reflecting the respective questions (“not important” to “very important,” “not good” to “very good,” “not threatening” to “very threatening”). We conducted independent sample *t*-test in R (R Development Core Team, 2017) to test if any significant changes have occurred between Study 1 and Study 2.

## 3 Results

The results and the comparison of our studies are presented in the following subsections. The results of Study 1 can also be found in our previous publication (Godbersen et al., 2020). In subsection 3.1, we will present the results regarding the subjective relevance of the attitude, subjective norm and perceived behavioral control, and the social spheres (RQ1). In subsection 3.2, the subjective qualities of the afore-mentioned elements are presented (RQ2). On this basis, we will show the potential and need for increasing or securing the effectiveness of the anti-Corona measures and derive norm strategies for their optimisation in Section 3.3 (RQ3). In subsection 3.4, we will present the results of our qualitative analysis regarding the reasons of our participants for positively or negatively evaluating the anti-Corona measures (RQ4). Finally, we will present the results of additionally examined variables.

### 3.1 Subjective relevance and (total) normed values of attitude, subjective norm and perceived behavioral control, and social spheres

Our first research objective is to determine the changes in the subjective relevance of the examined constructs (RQ1). To this end, we analyzed the empirical, normed and total normed values, which are represented in Table 1.

**Table 1:** Empirical, normed and total normed values.

Construct	Empirical value		Normed value		Total normed value	
	Study 1	Study 2	Study 1	Study 2	Study 1	Study 2
Attitude	**87.84** (SD = 16.79)	**81.53** (SD = 21.55)	0.39 (SD = 0.08)	0.40 (SD = 0.10)	0.39 (SD = 0.08)	0.40 (SD = 0.10)
Subjective norm	55.03 (SD = 28.67)	53.39 (SD = 28.11)	**0.23** (SD = 0.11)	**0.25** (SD = 0.11)	**0.23** (SD = 0.11)	**0.25** (SD = 0.11)
Perceived behavioral control	**83.57** (SD = 19.37)	**74.20** (SD = 24.08)	**0.37** (SD = 0.08)	**0.35** (SD = 0.08)	**0.37** (SD = 0.08)	**0.35** (SD = 0.08)
Close family (attitude)	**92.03** (SD = 15.12)	**87.64** (SD = 18.29)	**0.27** (SD = 0.05)	**0.28** (SD = 0.06)	**0.11** (SD = 0.04)	**0.11** (SD = 0.04)
Wider family and friends (attitude)	**86.52** (SD = 18.90)	**80.28** (SD = 23.36)	0.25 (SD = 0.03)	0.25 (SD = 0.04)	0.10 (SD = 0.02)	0.10 (SD = 0.03)
Colleagues at work (attitude)	**81.61** (SD = 22.87)	**74.51** (SD = 25.79)	**0.23** (SD = 0.05)	**0.23** (SD = 0.05)	0.09 (SD = 0.03)	0.09 (SD = 0.03)
Society in general (attitude)	**85.85** (SD = 18.07)	**79.33** (SD = 22.22)	0.25 (SD = 0.04)	0.25 (SD = 0.05)	0.10 (SD = 0.02)	0.10 (SD = 0.03)
Close family (subjective norm)	65.57 (SD = 29.13)	64.18 (SD = 29.23)	**0.30** (SD = 0.11)	**0.32** (SD = 0.11)	**0.07** (SD = 0.04)	**0.08** (SD = 0.04)
Wider family and friends (subjective norm)	**52.78** (SD = 29.71)	**49.00** (SD = 29.54)	0.23 (SD = 0.07)	0.23 (SD = 0.09)	0.05 (SD = 0.03)	0.06 (SD = 0.03)
Colleagues at work (subjective norm)	52.92 (SD = 29.65)	49.71 (SD = 28.99)	0.23 (SD = 0.09)	0.23 (SD = 0.10)	**0.05** (SD = 0.03)	**0.06** (SD = 0.04)
Society in general (subjective norm)	**52.18** (SD = 29.40)	**47.03** (SD = 28.86)	0.23 (SD = 0.10)	0.22 (SD = 0.11)	0.05 (SD = 0.03)	0.06 (SD = 0.04)
Close family (perceived behavioral control)	**86.85** (SD = 18.80)	**82.39** (SD = 22.07)	0.29 (SD = 0.08)	0.29 (SD = 0.09)	0.11 (SD = 0.04)	0.10 (SD = 0.04)
Wider family and friends (perceived behavioral control)	**74.33** (SD = 25.51)	**68.17** (SD = 27.61)	0.24 (SD = 0.06)	0.23 (SD = 0.08)	**0.09** (SD = 0.03)	**0.08** (SD = 0.03)
Colleagues at work (perceived behavioral control)	**74.31** (SD = 25.89)	**68.46** (SD = 26.67)	0.23 (SD = 0.06)	0.23 (SD = 0.07)	**0.09** (SD = 0.03)	**0.08** (SD = 0.03)
Society in general (perceived behavioral control)	**75.28** (SD = 24.78)	**69.20** (SD = 26.18)	0.24 (SD = 0.07)	0.24 (SD = 0.07)	**0.09** (SD = 0.03)	**0.08** (SD = 0.03)

*n* for Study 1 = 663; *n* for Study 2 = 577; bold numbers in green indicate significant increases with *p* < 0.05 and bold numbers in red indicate significant decreases with *p* < 0.05; the *p*-values can be found in Supplementary Table S1; own representation.

The empirical values of all of the examined constructs significantly decreased, with the exception of the subjective norm and its subordinated elements close family and colleagues at work.

The normed value of the subjective norm (willingness to fulfill the expectations of others) significantly increased, whilst the one of the perceived behavioral control (practicability of the anti-Corona measures) significantly decreased. The normed values of the close family for attitude (perceived protection from the coronavirus and its consequences) and subjective norm (willingness to fulfill the expectations of others) significantly increased. The normed value of colleagues at work significantly decreased for attitude (perceived protection from the coronavirus and its consequences).

The total normed values of close family for attitude (perceived protection from the coronavirus and its consequences) and subjective norm (willingness to fulfill the expectations of others) significantly increased. Furthermore, the total normed value of colleagues at work for subjective norm (willingness to fulfill the expectations of others) significantly increased. The total normed values of the wider family and friends, colleagues at work and society in general significantly decreased with regard to the perceived behavioral control (practicability of the anti-Corona measures).

All of the afore-mentioned changes are significant but cannot be considered as substantial. Furthermore, the normed and total normed values of the majority of constructs did not show significant changes during the COVID-19 pandemic and the relation of the constructs to each other remained stable, despite the significant changes mentioned above. This indicates that the structure of the subjective relevance of the people and, therewith, their expectations did not (fundamentally) change during the COVID-19 pandemic.

In both studies, the attitude (perceived protection from the coronavirus and its consequences) is more important to the people than the perceived behavioral control (practicability of the anti-Corona measures), which in turn has a higher subjective relevance than the subjective norm (willingness to fulfill the expectations of others). With regard to the social spheres, the close family is more important to the people than the wider family and friends, colleagues at work, and society in general.

### 3.2 Subjective quality of attitude, subjective norm and perceived behavioral control, and social spheres

**Figure 3:**
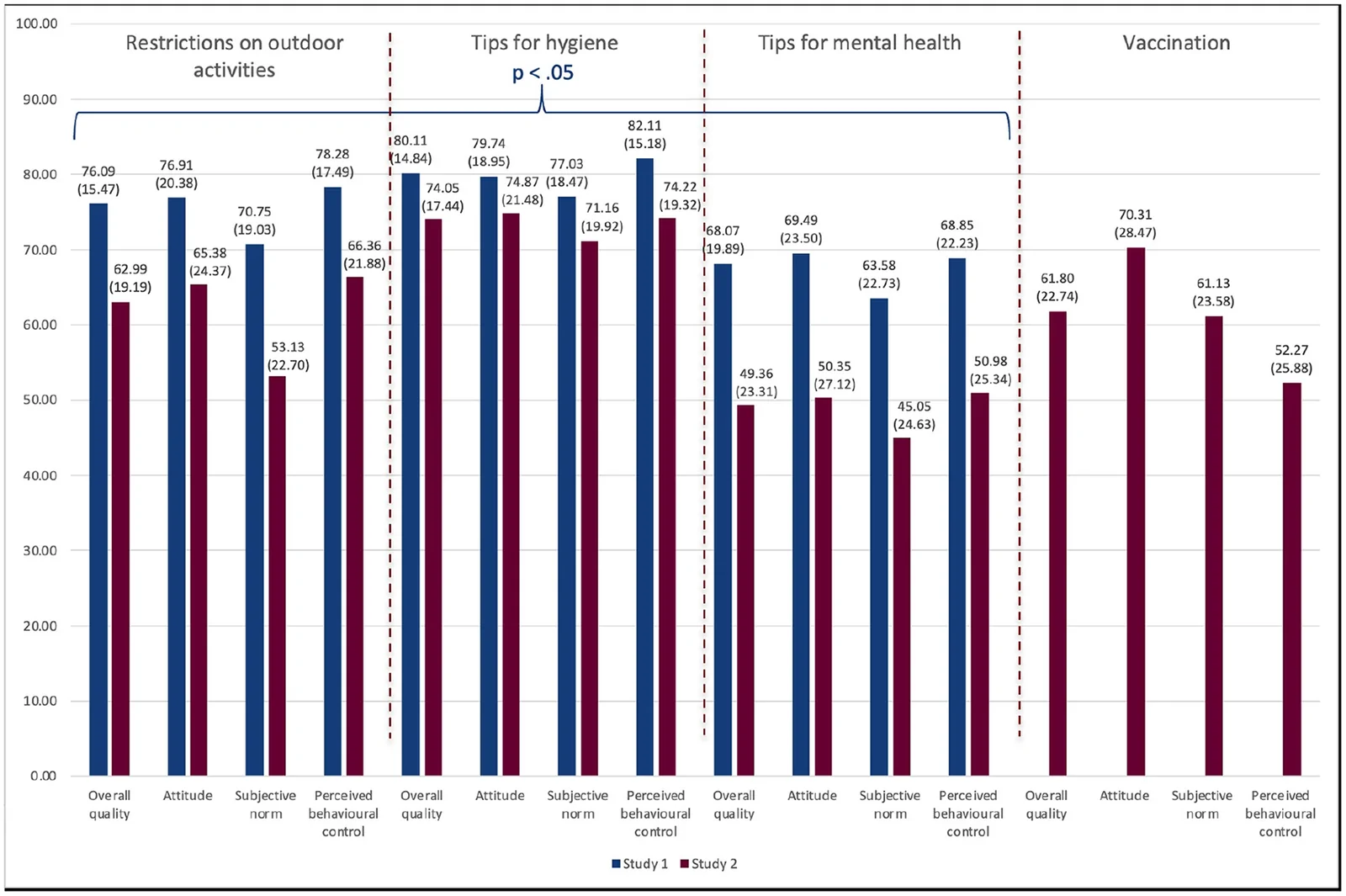
Calculated quality of anti-Corona measures and their subordinated constructs**—**attitude, subjective norm and perceived behavioral control (*n* for Study 1 = 663; *n* for Study 2 = 577; the p-values can be found in Supplementary Table S2 of the supplementary materials; own representation).

The second research objective refers to the development of the perceived quality of the anti-Corona measures restrictions on outdoor activities, tips for hygiene and tips for mental health during the pandemic, and the people's evaluation of the vaccination. The subjective qualities of the examined anti-Corona measures and the subjective qualities of the respective attitude (perceived protection from the coronavirus and its consequences), subjective norm (willingness to fulfill the expectations of others) and perceived behavioral control (practicability of the anti-Corona measures) are represented in Figure 3.

All subjective qualities for the restrictions on outdoor activities, tips for hygiene and tips for mental health and their subordinated elements (attitude, subjective norm and perceived behavioral control) significantly dropped during the COVID-19 pandemic. The decline in subjective quality can be considered substantial for the restrictions on outdoor activities and tips for mental health, as the former dropped from 76.09 to 62.99 and the latter from 68.07 to 49.36 on a scale from 0 (not good) to 100 (very good). In comparison, the decline in subjective quality of the tips for hygiene can be regarded as rather moderate by falling “only” from 80.11 to 74.05. The decrease in subjective qualities of the attitude, subjective norm and perceived behavioral control mirrors the development of the respective anti-Corona measure.

When only looking at the results of Study 2, tips for hygiene are evaluated best with an overall quality of 74.05 on a scale from 0 (not good) to 100 (very good), followed by restrictions on outdoor activities (62.99) and vaccination (61.80). The tips for mental health received the lowest quality rating with 49.36. The qualities for the attitude, subjective norm and perceived behavioral control follow a similar pattern for the restrictions on outdoor activities, tips for hygiene and tips for mental health. The attitude (perceived protection from the coronavirus and its consequences) and the perceived behavioral control (practicability of the anti-Corona measure) are evaluated on roughly the same level and are perceived with a better quality than the subjective norm (willingness to fulfill the expectations of others). A different picture emerges for the vaccination; here, the attitude (perceived protection from the coronavirus and its consequences) is perceived better than the subjective norm (willingness to fulfill the expectations of others), which, in turn, is assessed better than the perceived behavioral control (practicability of the anti-Corona measures).

All of the subjective qualities for the social spheres with regard to the restrictions on outdoor activities, tips for hygiene and tips for mental health decreased significantly during the COVID-19 pandemic, as can be seen in Table 2. A common pattern for the social spheres across the attitude (perceived protection from the coronavirus and its consequences), subjective norm (willingness to fulfill the expectations of others) and perceived behavioral control (practicability of the anti-Corona measure) and the four examined anti-Corona measures cannot be detected for Study 2.

**Table 2:** Empirical quality of the social spheres within the attitude, subjective norm and perceived behavioral control for the anti-Corona measures restrictions on outdoor activities, tips for hygiene, tips for mental health and vaccination.

Construct	Restrictions on outdoor activities		Tips for hygiene		Tips for mental health		Vaccination	
	Study 1	Study 2	Study 1	Study 2	Study 1	Study 2	Study 1	Study 2
Attitude: close family	**79.61** (SD = 22.62)	**67.80** (SD = 26.99)	**80.58** (SD = 22.08)	**75.58** (SD = 24.11)	**72.54** (SD = 25.08)	**52.87** (SD = 29.07)	**–**	70.39 (SD = 30.35)
Attitude: wider family and friends	**78.62** (SD = 22.21)	**65.20** (SD = 26.76)	**81.24** (SD = 20.45)	**74.73** (SD = 23.97)	**70.39** (SD = 25.09)	**50.51** (SD = 28.88)	**–**	68.49 (SD = 30.40)
Attitude: colleagues at work	**71.74** (SD = 27.36)	**60.90** (SD = 29.71)	**77.95** (SD = 22.95)	**73.13** (SD = 25.81)	**67.46** (SD = 26.49)	**50.56** (SD = 28.61)	**–**	68.70 (SD = 30.71)
Attitude: society in general	**75.67** (SD = 23.67)	**65.14** (SD = 26.95)	**78.22** (SD = 22.40)	**74.75** (SD = 23.08)	**66.12** (SD = 25.79)	**46.50** (SD = 28.57)	**–**	72.61 (SD = 27.81)
Subjective norm: close family	**77.93** (SD = 21.72)	**58.14** (SD = 29.05)	**81.48** (SD = 19.84)	**75.32** (SD = 23.16)	**67.60** (SD = 25.24)	**47.57** (SD = 28.63)	**–**	64.77 (SD = 31.96)
Subjective norm: wider family and friends	**70.34** (SD = 23.31)	**49.32** (SD = 27.49)	**77.96** (SD = 20.44)	**70.17** (SD = 24.43)	**64.39** (SD = 24.91)	**43.83** (SD = 27.19)	**–**	58.35 (SD = 29.32)
Subjective norm: colleagues at work	**67.24** (SD = 25.61)	**51.64** (SD = 27.48)	**75.86** (SD = 22.87)	**71.20** (SD = 23.85)	**61.72** (SD = 25.31)	**45.36** (SD = 26.70)	**–**	62.43 (SD = 26.88)
Subjective norm: society in general	**65.31** (SD = 20.92)	**46.85** (SD = 23.51)	**71.70** (SD = 21.28)	**63.33** (SD = 22.55)	**59.57** (SD = 23.87)	**40.82** (SD = 24.68)	**–**	55.36 (SD = 20.80)
Perceived behavioral control: close family	**82.24** (SD = 23.75)	**63.48** (SD = 32.15)	**82.82** (SD = 22.55)	**72.12** (SD = 27.78)	**75.16** (SD = 25.01)	**56.62** (SD = 30.02)	**–**	55.35 (SD = 30.84)
Perceived behavioral control: wider family and friends	**84.29** (SD = 23.37)	**68.40** (SD = 30.68)	**87.18** (SD = 17.87)	**77.17** (SD = 23.79)	**69.95** (SD = 25.31)	**51.34** (SD = 28.77)	**–**	50.25 (SD = 28.94)
Perceived behavioral control: colleagues at work	**70.84** (SD = 31.40)	**66.46** (SD = 32.06)	**80.60** (SD = 23.61)	**75.05** (SD = 26.08)	**65.23** (SD = 27.23)	**49.71** (SD = 28.37)	**–**	52.55 (SD = 28.69)
Perceived behavioral control: society in general	**73.18** (SD = 24.13)	**67.46** (SD = 26.69)	**76.50** (SD = 22.85)	**73.16** (SD = 22.87)	**61.36** (SD = 27.14)	**44.53** (SD = 27.77)	**–**	50.35 (SD = 25.68)

*n* for Study 1 = 663; *n* for Study 2 = 577; bold numbers indicate significant decreases with *p* < 0.05; the *p*-values can be found in Supplementary Table S3; own representation.

### 3.3 Norm strategies for optimizing anti-Corona measures

With regard to our third research objective, we examined how the potential and need for optimizing the restrictions on outdoor activities, tips for hygiene and tips for mental health and, therewith, the derived norm strategies changed over the course of the COVID-19 pandemic (RQ3.1). Furthermore, we aim to determine the potential and need for optimizing the vaccination from the people's perspective and the respective norm strategies in Study 2 (RQ3.2).

#### 3.3.1 Norm strategies for the restrictions on outdoor activities, tips for hygiene and tips for mental health

**Figure 4:**
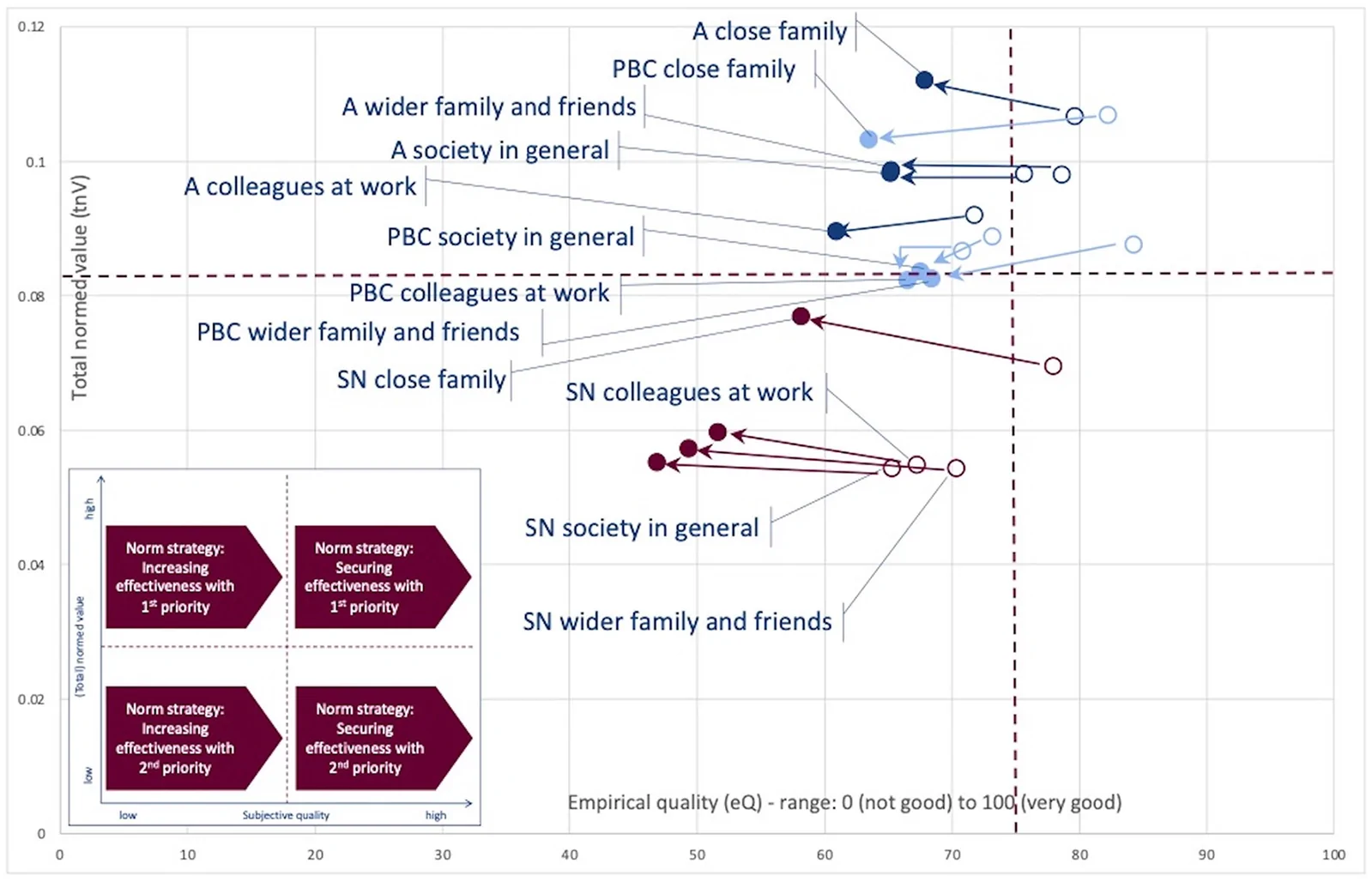
Empirical quality and total normed values of the attitude (A), subjective norm (SN) and perceived behavioral (PBC) within the social spheres regarding restrictions on outdoor activities (empty dots represent Study 1 with *n* = 663; filled dots represent Study 2 with *n* = 577; own representation).

**Figure 5:**
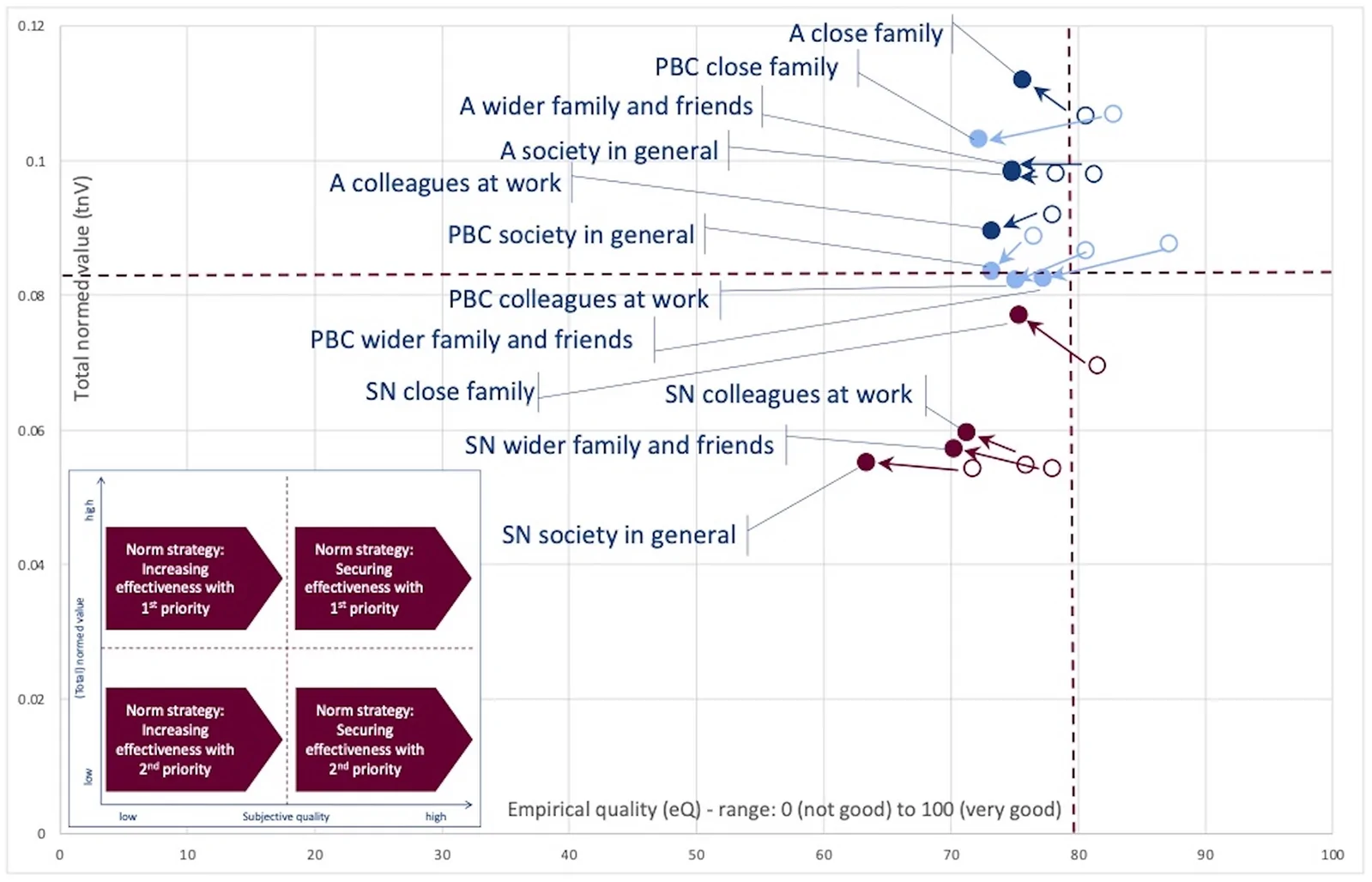
Empirical quality and total normed values of the attitude (A), subjective norm (SN) and perceived behavioral (PBC) within the social spheres regarding tips for hygiene (empty dots represent Study 1 with *n* = 663; filled dots represent Study 2 with *n* = 577: own representation).

**Figure 6:**
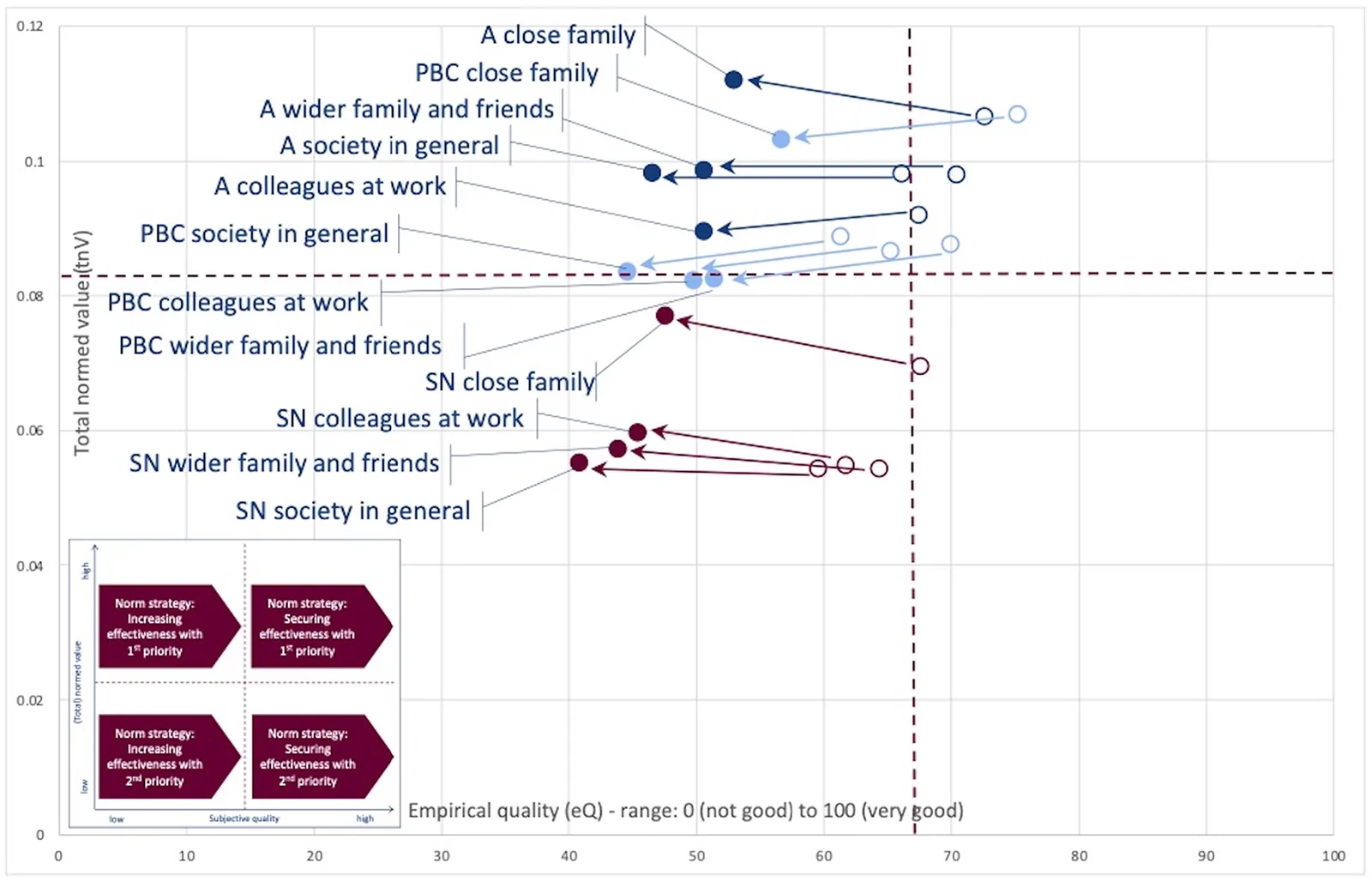
Empirical quality and total normed values of the attitude (A), subjective norm (SN) and perceived behavioral (PBC) within the social spheres regarding tips for mental health (empty dots represent Study 1 with *n* = 663; filled dots represent Study 2 with *n* = 577: own representation).

The total normed values (tnV) and the subjective quality (eQ) of the attitude, subjective norm and perceived behavioral control for the four social spheres (close family, wider family and friends, colleagues at work, and society in general) with regard to the restrictions on outdoor activities are presented in the form of a matrix in Figure 4, with regard to tips for hygiene in Figure 5 and with regard to tips for mental health in Figure 6. In all of the three matrices, the separating line between the quadrants regarding the subjective quality is based on the results of Study 1, as the first evaluation at the beginning of the COVID-19 pandemic can be understood as the mental reference for the people.

Based on the results of Study 2, the effectiveness of the restrictions on outdoor activities, tips for hygiene and tips for mental health should be increased with a high priority with regard to the following elements (Figures 4–6, top left quadrant):

- Attitude within the social sphere of the close family.
- Attitude within the social sphere of the wider family and friends.
- Attitude within the social sphere of the colleagues at work.
- Attitude within the social sphere of the society in general.
- Perceived behavioral control within the social sphere of the close family.
- Perceived behavioral control within the social sphere of the society in general.

With a lower priority, the effectiveness of the restrictions on outdoor activities, tips for hygiene and tips for mental health should be increased with regard to the following elements (Figures 4–6, bottom left quadrant):

- Perceived behavioral control within the social sphere of the wider family and friends.
- Perceived behavioral control within the social sphere of the colleagues at work.
- Subjective norm within the social sphere of the close family.
- Subjective norm within the social sphere of the wider family and friends.
- Subjective norm within the social sphere of the colleagues at work.
- Subjective norm within the social sphere of the society in general.

We showed in the previous section that the perceived qualities of all of the elements of the hypothesized model significantly and substantially declined for all of the anti-Corona measures, assessed in Study 1 and Study 2, during the COVID-19 pandemic. Thus, the need for optimizing the restrictions on outdoor activities, tips for hygiene and tips for mental health with regard to the examined elements also changed and, therewith, the advised norm strategies, as can be seen in Figures 4–6. The attitude (perceived protection from the coronavirus and its consequences) and the perceived behavioral control (practicability of the anti-Corona measure) within the social spheres of the close family as well as the wider family and friends were positioned in the top right quadrant for all of the three anti-Corona measures in Study 1, meaning that the effectiveness of these measures should have been secured with a high priority in this regard. During the COVID-19 pandemic, these elements were evaluated with a lower quality in Study 2 and moved to the top left quadrant (Study 2), which means the referring effectiveness has to be increased. Similarly, the following elements showed a low need for increasing their effectiveness, as they were positioned in the top right quadrant in Study 1, but moved to the left quadrants during the COVID-19 pandemic: the attitude (perceived protection from the coronavirus and its consequences) within the social sphere of the society in general with regard to restrictions on outdoor activities, the attitude (perceived protection from the coronavirus and its consequences) within the social sphere of the colleagues at work with regard to tips for mental health, and the perceived behavioral control (practicability of the anti-Corona measures) within the social sphere of colleagues at work with regard to tips for hygiene.

#### 3.3.2 Norm strategies for the vaccination

**Figure 7:**
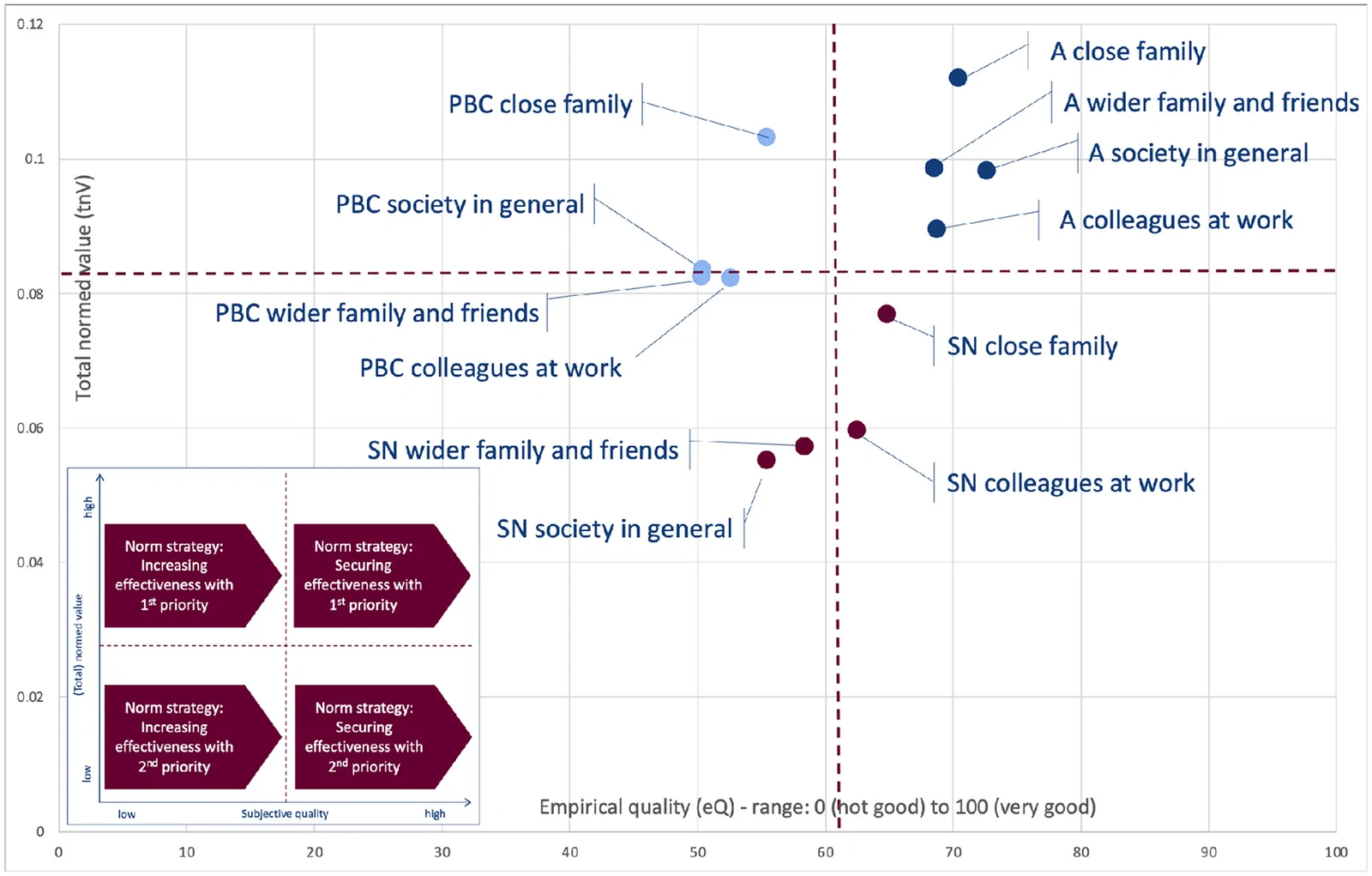
Empirical quality and total normed values of the attitude (A), subjective norm (SN) and perceived behavioral (PBC) within the social spheres regarding vaccination (data was collected only in Study 2 with *n* = 577; own representation).

The total normed values (tnV) and the subjective quality (eQ) of the attitude, subjective norm and perceived behavioral control for the four social spheres (close family, wider family and friends, colleagues at work, and society in general) with regard to the vaccination are presented in the form of a matrix in Figure 7.

Based on the results of Study 2, the effectiveness of the vaccination should be increased with a high priority with regard to the following elements (Figure 7, top left quadrant):

- Perceived behavioral control within the social sphere of the close family.
- Perceived behavioral control within the social sphere of the society in general.

With a lower priority, the effectiveness of the vaccination should be increased with regard to the following elements (Figure 7, bottom left quadrant):

- Perceived behavioral control within the social sphere of the wider family and friends.
- Perceived behavioral control within the social sphere of the colleagues at work.
- Subjective norm within the social sphere of the wider family and friends.
- Subjective norm within the social sphere of the society in general.

The effectiveness of the vaccination should be mainly secured with a higher priority with regard to the following elements (Figure 7, top right quadrant):

- Attitude within the social sphere of the close family.
- Attitude within the social sphere of the wider family and friends.
- Attitude within the social sphere of the colleagues at work.
- Attitude within the social sphere of the society in general.

With a lower priority, the effectiveness of the vaccination should be mainly secured with regard to the following elements (Figure 7, bottom left quadrant):

- Subjective norm within the social sphere of the close family.
- Subjective norm within the social sphere of the colleagues at work.

### 3.4 Qualitative analysis of people's evaluation of anti-Corona measures

The fourth objective of our research is to discover the subjective reasons for the people's positive and negative evaluation of anti-Corona measures. To this end, we asked the participants in an open question to give their personal rationale behind their quantitative assessment of the restrictions on outdoor activities, tips for hygiene, tips for mental health and vaccination. As described in the methodology section, we predefined three categories for our analysis: positive evaluation, criticism and ambiguity/not applicable. The subordinated categories for the positive evaluation of anti-Corona measures, which we developed inductively from the participants' answers, and their frequencies as percentage of participants are represented in Table 3. The respective subcategories and frequencies for criticism of the anti-Corona measures can be found in Table 4. Examples for the original German statements can be found in Supplementary Tables S4–S7.

**Table 3:** Reasons for the positive evaluation of the anti-Corona measures with main categories and subcategories and the associated frequencies in relation to the total number of participants for restrictions on outdoor activities, tips for hygiene, tips for mental health and vaccination.

Main and subcategories	Restrictions on outdoor activities	Tips for hygiene	Tips for mental health	Vaccination
Main category: positive evaluation of measures in general				
Measure is effective and/or makes sense in general	42.46%	50.43%	20.80%	39.34%
Measure is effective because of case numbers, studies, experts and/or official approval	3.64%	1.56%	0.00%	4.68%
Measure is the best known solution	1.04%	0.87%	0.00%	5.37%
Main category: poisitive evaluation of measures with a goal-orientation				
Measure serves the individual protection from the virus	0.00%	0.00%	0.00%	5.55%
Measure helps to protect the society, others and/or risk groups	1.39%	1.39%	0.00%	4.85%
Measure is a mean to quickly return to a normal life	0.35%	0.00%	0.00%	14.38%
Main category: positive evaluation of measures with a focus on execution				
Measure is well explained and/or easy to understand	0.00%	6.93%	0.00%	0.00%
Measure is practicable and/or not to restrictive in one's daily life	0.00%	14.21%	0.17%	0.00%
Measure is good in general and not only during the pandemic	0.00%	28.42%	1.56%	0.00%
Measure is necessary because appealing to reason does/did not work	2.77%	0.00%	0.00%	0.00%

Data were collected only in Study 2 with *n* = 577; own representation.

**Table 4:** Reasons for criticism of the anti-Corona measures with main categories and subcategories and the associated frequencies in relation to the total number of participants for restrictions on outdoor activities, tips for hygiene, tips for mental health and vaccination.

Main and subcategories	Restrictions on outdoor activities	Tips for hygiene	Tips for mental health	Vaccination
Main category: criticism of measures in general				
Measure is not effective and/or does not make sense in general	5.55%	1.21%	14.90%	3.29%
Measure is not effective because of numbers and/or studies	2.25%	0.00%	0.00%	0.00%
Measure is not sufficiently researched, tested and/or thought trough	3.99%	0.00%	0.00%	11.09%
Main category: criticism of the extend of measures				
Measure should be strengthened/enhanced and/or taken earlier	10.05%	0.52%	14.73%	0.00%
Measure is too much, too strict and/or too long	2.60%	0.00%	0.00%	0.00%
15.6-7.4,-14.3498ptThere should have been one hard lockdown and then the society should have been opened again	3.12%	0.00%	0.00%	0.00%
Main category: criticism of specific rules				
Specific rules of a measure are not effective/do not make sense	18.72%	2.08%	0.00%	0.00%
Rules contradict themselves and/or are inconsistent	5.89%	0.17%	0.00%	0.00%
Rules should be regionalised depending on case numbers	0.17%	0.00%	0.00%	0.00%
Rules should be consistent and uniform all over the country	1.73%	0.00%	0.00%	0.00%
Main category: criticism of measures regarding their execution				
Knowledge/transparency of measure is low	1.91%	1.04%	12.82%	3.12%
Rules are difficult to accept/follow and/or are tiering/exhausting oder time	3.99%	5.72%	14.04%	0.00%
Others do not sufficiently obey the rules and/or rules should be enforced more strictly	9.19%	8.49%	0.00%	0.00%
Specific rules of a measure are not effective/do not make sense	18.72%	2.08%	0.00%	0.00%
Main category: criticism of measures regarding side-effects				
The vaccination process is poorly organized and/or executed	0.00%	0.00%	0.00%	4.68%
Measure affects quality of life and/or mental health	9.01%	0.69%	0.00%	0.00%
Measure is a restriction of freedom	4.16%	0.00%	0.00%	0.00%
Measure causes economic damage	7.45%	0.00%	0.00%	0.00%
Measure may cause unkown (long-term) side-effects	0.00%	0.00%	0.00%	9.53%
Main category: criticism of the individuality and specificity of measures				
A general approach does not work and/or measure should be individualized	0.00%	0.00%	8.32%	0.00%
Measure is too superficial, not specific enough and/or no help for severely affected people	0.00%	0.00%	14.04%	0.00%
Measure cannot compensate the social situation	0.00%	0.00%	9.36%	0.00%

Data were collected only in Study 2 with *n* = 577; own representation.

Statements we assigned to the category of ambiguity/not applicable are “I am in two minds” or “I do not have a reason.”

The positive evaluation of the anti-Corona measures consists of three main categories:

- Positive evaluation in general.
- Positive evaluation with a goal-orientation.
- Positive evaluation with a focus on execution.

The main category positive evaluation in general has three subcategories. Some participants indicated a general agreement with the anti-Corona measures without giving specific reasons (typical statement: “I am convinced of that”); meaning an anti-Corona measure is regarded effective and/or makes sense in general from the participants point of view. Secondly, participants positively evaluated an anti-Corona measure because the measure is regarded as being effective because of case numbers, studies, experts and/or official approval (typical statement: “the decreasing infection numbers show that this is right”). Thirdly, participants stated that a measure is the best-known solution (typical statement: “in my opinion, there is no other option to reduce the numbers”).

The main category positive evaluation with a goal-orientation consists of three subcategories. The first subcategory of reasons for positively evaluating an anti-Corona measure refers to the individual protection from the virus (typical statement: “I think highly of it because we are protected by a vaccination in the long-run”). The second reason within this main category focuses on the protection of the society, others and/or risk groups (typical statement: “the tips are important so that you also protect others from the virus”). Thirdly, participants regarded an anti-Corona measure as a mean to quickly return to a normal life (typical statement: “it will allow a normal daily life”).

For the main category positive evaluation of anti-Corona measures with a focus on execution, there are four subcategories of reasons. It is positive for participants if measures are well explained and/or simple to understand (typical statement: “sufficiently explained and makes sense and comprehensible”). Furthermore, it is important to the participants that an anti-Corona measure is practicable and/or not too restrictive in their daily lives (typical statement: “simple and easy to realize”). It seems to be appealing to some participants if an anti-Corona measure is perceived as being good in general und not only during the pandemic (typical statement: “good tips; universal; also useful outside of the pandemic”). Finally, restrictive anti-Corona rules are positively evaluated by some of the participants because, in their view, appealing to reason does/did not work (typical statement: “because appealing to reason did not work, such restriction have to be taken”).

Of the afore-described ten subcategories, the following subcategories were mentioned most often in the context of a positive evaluation of anti-Corona measures:

- Measure is effective and/or makes sense in general.
- Measure is a mean to quickly return to a normal life.
- Measure is practicable and/or not too restrictive in one's daily life.
- Measure is good in general and not only during the pandemic.

Criticism on anti-Corona measures can be structured in six main categories:

- Criticism in general.
- Criticism of the extend of measures.
- Criticism of specific rules.
- Criticism regarding the execution of measures.
- Criticism regarding side-effects of measures.
- Criticism of the individuality and specificity of measures.

The main category criticism in general has three subcategories. Participants stated that a measure is not effective and/or does not make sense in general, without giving specific reasons (typical statement: “I personally do not find the tips so helpful”). The measure might be regarded as not effective because of numbers and/or studies (typical statement: “no proven reduction of infection numbers”). Lastly, criticism stems from the opinion that a measure is not sufficiently researched, tested and/or thought through (typical statement: “no scientific approach but acting because of fear; it lacks innovations and a long-term perspective”).

The main category criticism of the extend of measures has three subcategories. On the one hand, participants think that the measures should be strengthened, enhanced and/or taken earlier (typical statement: “measures should be tougher”). On the other hand, participants think a measure is too much, too strict and/or too long (typical statement: “I think the restrictions on outdoor activities are to exaggerated and to restrictive”). Additionally, some state that there should have been one hard lockdown and then the society should have been opened again (typical statement: “better short and crisp instead of long and mild”).

The main category criticism of specific rules has four subcategories. Some participants are of the opinion that specific rules or characteristics of measure are not effective and/or do not make sense (typical statement: “[referring to the restrictions on outdoor activities] is Corona more active after 21:00hrs? I do not think so!”). Other participants think that the rules contradict themselves and/or are inconsistent (typical statement: “The restrictions on outdoor activities contradict themselves and, quite often, do not make sense”). In the context of the federal structure of Germany, the participants either criticize that the rules should be regionalised depending on case numbers (typical statement: “the measures must be different in the federal states, or, at least, there should only be restrictions where the case numbers are high”) or criticize that the rules should be consistent and uniform all over the country (typical statement: “the realization should be the same way in all of the federal states”).

The main category criticism regarding the execution of measures has four subcategories. It is criticized that the knowledge/transparency of a measure is low (typical statement: “about this, there is too few information for the citizens”). Furthermore, some participants regard it as difficult to accept/follow the rules and/or find the rules tiering/exhausting over time (typical statement: “some measures are difficult to realize”). Another aspect of criticism from some participants' perspective is that others do not sufficiently obey the rules and/or the rules should be enforced more strictly (typical statement: “partly, I would prefer that the restrictions on outdoor activities are controlled and sanctioned in a tougher way”). With regard to the vaccination, it is stated that the vaccination process is poorly organized and/or executed (typical statement: “the process of vaccinating is not organized enough in Germany”).

The main category criticism regarding side-effects of measures has four subcategories. Participants are of the opinion that measures negatively affect the quality of life and/or mental health (typical statement: “because of the restrictions, my psyche has strongly decreased, also amongst my friends”). Other participants regard the restriction of freedom as critical (typical statement: “here, the freedom is restricted too much”). A damaging effect on the economy is also seen as problematic (typical statement: “at the moment, the measures are too hard so that many go into bankruptcy”). With regard to the vaccination, it is criticized that (long-term) side-effects may occur (typical statement: “it is not enough known about aftereffects”).

The main category individuality and specificity of measures has three subcategories. A lack of Individuality and specificity is criticized with regard to the tips for mental health. It is stated that a general approach does not work and/or a measure should be individualized (typical statement: “too general, everybody needs his own tips”). Furthermore, some participants criticize that the tips for mental health are too superficial, not specific enough and/or no help for severely affected people (typical statement: “nice approach, but if someone really suffers from anxiety or similar”). Finally, it is believed by some that this measure cannot compensate the social situation (typical statement: “these tips do not support my mental health, the only thing that helps is social contacts”).

Of the afore-described 21 subcategories, the following subcategories were mentioned most often in the context of criticizing the anti-Corona measures:

- Measure is not effective and/or does not make sense in general.
- Measure is not sufficiently researched, tested and/or thought trough.
- Measure should be strengthened/enhanced and/or taken earlier.
- Specific rules of a measure are not effective/do not make sense.
- Knowledge/transparency of measure is low.
- Rules are difficult to accept/follow and/or are tiering/exhausting over time.
- Others do not sufficiently obey the rules and/or rules should be enforced more strictly.
- Measure is too superficial, not specific enough and/or no help for severely affected people.

### 3.5 Perceived information level, information sources and perceived threat

Apart from our main quantitative and qualitative analysis, we examined the perceived quality of the information level about the COVID-19 pandemic, the subjective quality of information by the government and researchers or research institutes, the relevance of information sources, and the perceived threat of COVID-19.

**Figure 8:**
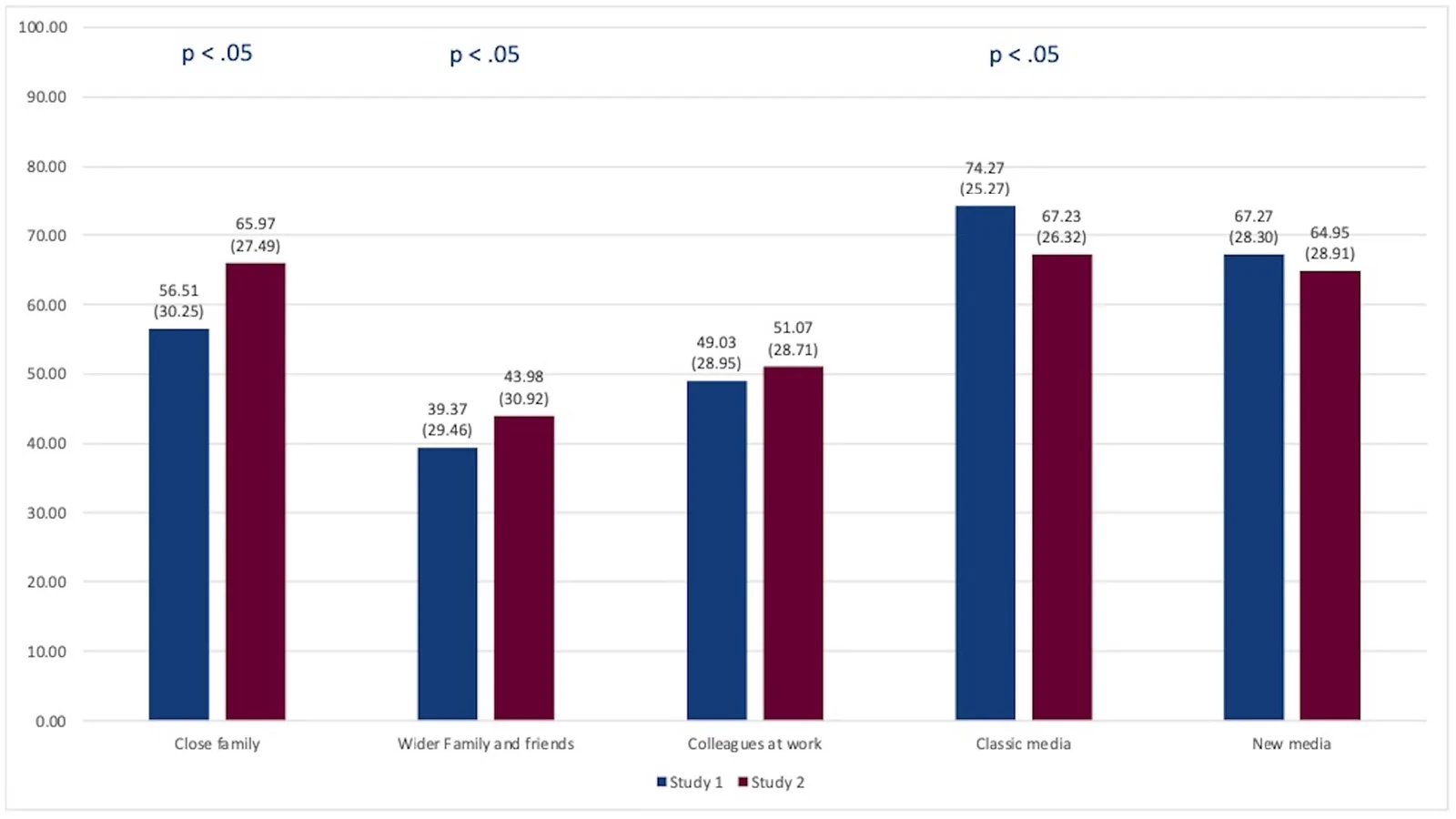
Relevance of information sources on a scale from 0 (not important) to 100 (very important; *n* for Study 1 = 663; *n* for Study 2 = 577; own representation).

**Figure 9:**
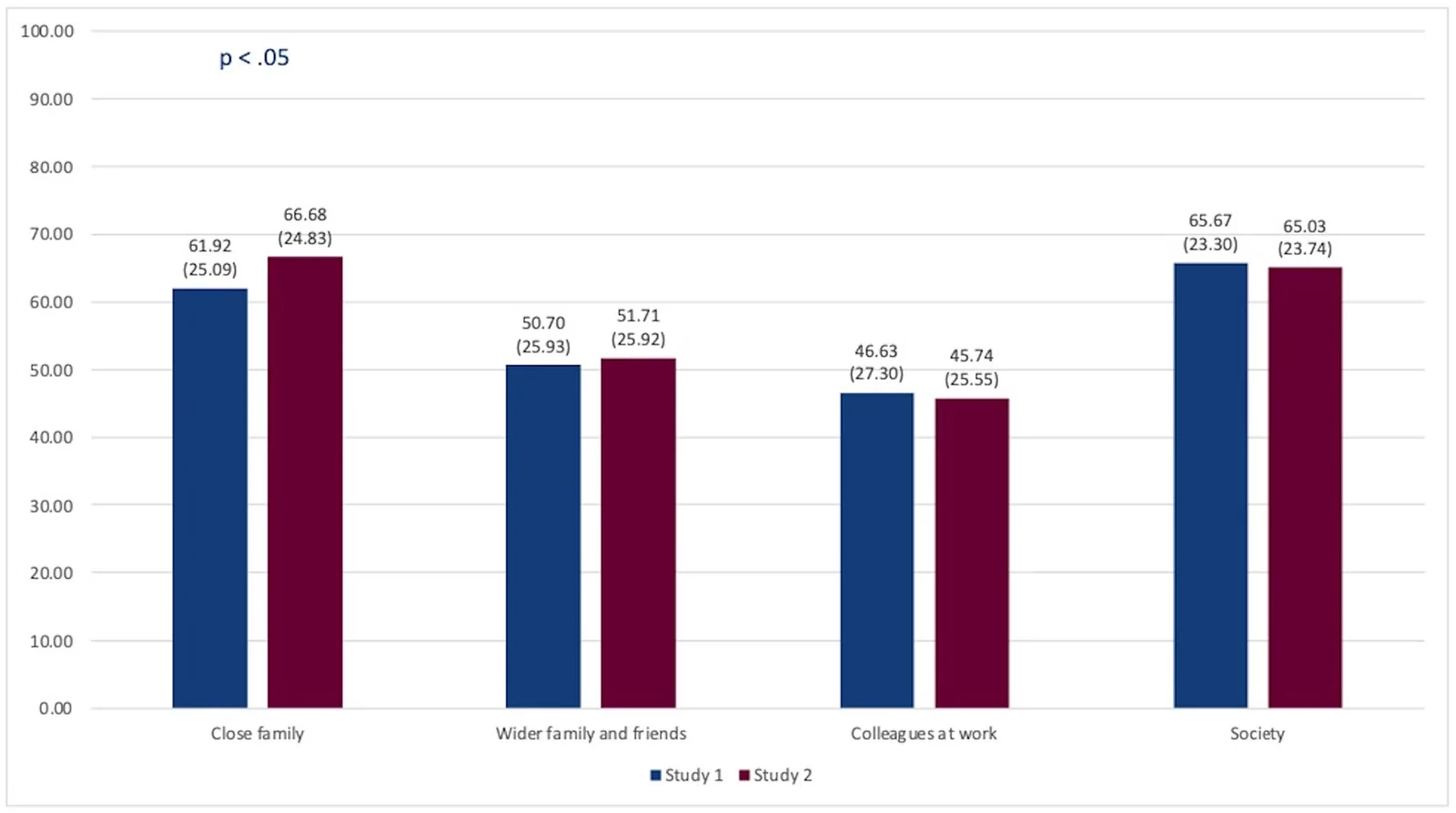
Perceived threat for the social spheres on a scale from 0 (not threatening) to 100 (very threatening; *n* for Study 1 = 663; *n* for Study 2 = 577; own representation).

The perceived quality of the information level significantly declined during the COVID-19 pandemic from 69.16 (SD = 21.03) in Study 1 to 64.12 (SD = 21.33) in Study 2 on a scale from 0 not good to 100 very good (*p* < 0.05). On the same scale, the perceived quality of information provided by the government (Study 1: 64.30, SD = 23.66 and Study 2: 57.19, SD = 24.11) and researchers or research institutes (Study 1: 69.84, SD = 24.49 and Study 2: 57.08, SD = 25.72) declined significantly (*p* < 0.05). The relevance (measured on a scale from 0 not important to 100 very important) of the close family and the wider family and friends as a source of information increased significantly from Study 1 to Study 2, whilst the relevance of classic media significantly decreased, as can be seen in Figure 8. The perceived threat level of COVID-19 (measured on a scale from 0 not threatening to 100 very threatening) significantly increased with regard to the close family from Study 1 to Study 2, whilst significant changes could not be detected for the other examined social spheres, as shown in Figure 9.

## 4 Discussion

In the following two subsection, we will discuss the implications of our research, especially for designing, executing and communicating measures to counter a severe crisis from the people's perspective, outline the limitations of our studies and give an outlook on research that could follow our work.

### 4.1 Implications

The analysis of the empirical and (total) normed values revealed that the structure of the subjective relevance with regard to the protection from the coronavirus and its consequences (attitude), the willingness to fulfill the expectations of others (subjective norm) and the practicability of the anti-Corona measures (perceived behavioral control), and the social spheres did not fundamentally change during the COVID-19 pandemic. This indicates that the people have relatively stable and consistent expectations on how a government and other institutions involved should deal with a severe crisis like the COVID-19 pandemic.

In both of our studies, the perceived protection from COVID-19 (attitude) is more important to the people than the practicability of the anti-Corona measures (perceived behavioral control), which, in turn, is substantially more important than the willingness to fulfill the expectations of others (subjective norm). This means that policy makers should firstly focus on how measures can provide a utility to the people; in the case of a threat like the coronavirus, how the measures can protect the people. Secondly, the policy makers should make sure that the people can relatively easily integrate these measures into their day-to-day lives. Thirdly, policy makers are advised not to realize campaigns that incorporate social pressure. When looking at the social spheres, both studies show that, in the context of measures in a severe crisis, the close family is more important to the people than the wider family and friends, colleagues at work, and society in general, whose subjective relevance is on a comparable level. The emphasis the people place on the small family (in times of crisis) is also supported by the fact that the perceived threat significantly rose for the close family, as shown in the previous section. This indicates that policy makers should primarily focus on how the nuclear family can benefit from measures, implemented in a severe crisis, and how these measures can be best integrated in family lives. These conclusions match the ones of our publication of the first Study at the beginning of the COVID-19 pandemic (Godbersen et al., 2020).

In the previous section, we could show that the quality of all of the elements of the hypothesized model significantly declined for the restrictions on outdoor activities, tips for hygiene and tips for mental health during the COVID-19 pandemic. Furthermore, the norm strategies for improving the three afore-mentioned anti-Corona measures demand an increase in effectiveness for all of the elements of the hypothesized model in Study 2, whereas the norm strategy of securing effectiveness was found for some of these elements in Study 1. There are two possible explanations for this decline in perceived quality.

Firstly, wear-down effects, might be responsible for the people evaluating the anti-Corona measures on a worse quality level roughly after a year into the COVID-19 pandemic. In the context of a severe and enduring crisis, people might get worn down, as they cannot see an end of the adverse situation. Then, the counter measures can be as good and effective as it gets but the people attribute the lack of positive results to these measures. This effect can also be explained through chronic stress causing depressive symptoms (McGonagle and Kessler, 1990), as a year of perceived threat of the coronavirus and the perceived restrictions of the anti-Corona measures and their side-effects can lead to stress; the possibly resulting negative mood may lead to more negative evaluations of the anti-Corona measures.

Secondly, the reason for the subjective quality decline of the anti-Corona measures might be that the measures were actually poorly designed, executed and communicated regarding the people's perspective. Indicators for that can be seen in the complexity of the anti-Corona measures and the sluggish start of the vaccination campaign, as described in Section 1.1. Furthermore, the governmental communication campaigns to promote vaccination incorporated, at least in parts, some form of social pressure, which is ill-advised, as we pointed out above. The argument, made in this paragraph, is further supported by the decline of our additionally collected variables during the course of the COVID-19 pandemic, indicating that the people felt they can rely less on publicly available information after a year into the pandemic. In this context, the people feel less informed in general and by governmental and research sources 1 year into the pandemic than at the start, as shown in the previous section. This finding is especially relevant and even concerning, as Dohle et al. (2020) show that trust in politics and science are important predictors of accepting and adopting anti-Corona measures. Furthermore, the information about COVID-19 from the close family and family and friends became more important, whilst the relevance of classic media decreased. The second explantion for declining quality ratings is supported by the findings and rationale of Diehl and Wolter (2021), i.e., trust in and support for policies and institutions do not change, unless governmental actions have clear and substantial effects on the self-interests of the people—which seemed to be the case during the COVID-19 pandemic.

Regardless of which explanation is considered true for the anti-Corona measures' decline in quality ratings, policy makers are advised to substantially re-think the design, execution and communication of measures countering a severe crisis in the future. The need to employ high quality policies to counter severe crises is supported by Jaschke et al. (2023) who found that satisfaction with governmental measures increases the adherences to anti-Corona measures. Furthermore, it should be mentioned that, from the people's perspective, the vaccination is not the panacea for all of the problems of the COVID-19 pandemic, as what it was often portrayed. The vaccination is not the best evaluated anti-Corona measure, but ranks in third place roughly on the same quality level as the restrictions on outdoor activities. The tips for hygiene are evaluated better and the tips for mental health are evaluated worse. This indicates that the people evaluate concrete and “tangible” measures with a short-term perspective (tips for hygiene) better than rather abstract and “intangible” measures with long-term orientation (vaccination and tips for mental health).

From our qualitative analysis, we can deduce an approach on how to design, execute and communicate counter-measures in case of a severe crisis from the people's perspective. The most often mentioned reasons for the positive evaluation of the anti-Corona measures and most often stated criticism, as documented in the previous section, form the basis for this approach.

From the people's perspective, (governmental) measures to counter a severe crisis should have clear objectives and lead to substantial effects, which can be deduced from the following positive and critical evaluations of the anti-Corona measures:

- Measure is effective and/or makes sense in general (positive evaluation).
- Measure is a means to quickly return to a normal life (positive evaluation).
- Measure is not effective and/or does not make sense in general (criticism).
- Measure should be strengthened/enhanced and/or taken earlier (criticism).
- Specific rules of a measure are not effective / do not make sense (criticism).

Furthermore, it is important to the people that such measures are practicable and can be integrated in their “real” daily lives with relative ease, which can be deduced from the following positive and critical evaluations of the anti-Corona measures:

- Measure is practicable and/or not too restrictive in one's daily life (positive evaluation).
- Rules are difficult to accept/follow and/or are tiering/exhausting over time (criticism).
- Others do not sufficiently obey the rules and/or rules should be enforced more strictly (criticism).

- Measure is too superficial, not specific enough and/or no help for severely affected people (criticism).

At the same time, the measures countering a severe crisis should be based on facts and sciences, which can be deduced from the following critical evaluation of the anti-Corona measures:

- Measure is not sufficiently researched, tested and/or thought trough (criticism).

Finally, it is of relevance to the people that (governmental) counter-measures in a crisis are comprehensively and comprehensibly explained, which can be deduced from the following positive and critical evaluations of the anti-Corona measures:

- Measure is good in general and not only during the pandemic (positive evaluation).
- Knowledge/transparency of measure is low (criticism).

In summary and from the people's perspective, (governmental) measures to counter a severe crisis should have clear objectives and aim for substantial and positive effects for the people; they should be practicable and easy to integrate in the “real” day-to-day lives of the people; they should be based on solid facts and reliable scientific results; and they should be comprehensively and comprehensibly explained. This approach culminates in the so often used and misused slogan of “keep it simple and strong”; to clarify, we advocate, based on our data, for simple and effective solutions, which does not mean that these solutions are easy to design, execute and communicate.

### 4.2 Limitations and outlook

Our findings suggest that “simple (but not easy) and strong” solutions are best during severe crises. However, we must admit that we only examined one crisis, i.e. the COVID-19 pandemic, and, thus, cannot generalize this conclusion to all possible crises with certainty. Against this backdrop, it seems fruitful to examine the outcomes of past crises and their respective solutions. Such research would most likely go beyond the boundaries of psychology and should, therefore, involve at least historians and/or researchers from related disciplines.

Within the discipline of psychology, it might be interesting to investigate what psychological effects “simple” and “complex” solutions to crises and problems have on people. Experiments with, presumedly hypothetical, “simple” and “complex” solutions as stimuli spring to mind. In such experiments, the possible effects of rather personality traits and states on the evaluation of measures against severe crises could also be examined.

We collected our data at the beginning of the COVID-19 pandemic and after 1 year into the pandemic, when there was still no certain end to the pandemic in sight. As perceptions could change in hindsight and things could, then, be evaluated with a positive (or negative) bias, a follow-up study with the same design and measurement instruments might uncover interesting results.

Finally, the data of our studies were collected only in Germany. Even though it might be a fair assumption that people in general, at least in developed countries, would evaluate the anti-Corona measures similarly to Germans, we cannot rule out cultural influences. Thus, multi-country studies regarding (governmental) measures to counter (severe) crises might be useful to find a more differentiated solutions that can help people best all over the world.

In this context, future research should consider broader and more representative samples. Our samples constisted exclusively of part-time business psychology students. As these students work in regular jobs and study on the side, they represent “normal” people rather than typical students. However, our participants are younger, in earlier stages of their careers and more educated than the average population, which can have an impact on their evaluation of the anti-Corona measures. Thus, the generalization of our findings is not invalidated but might be limited to some extend.

## Data Availability

The raw data supporting the conclusions of this article will be made available by the authors, without undue reservation.
