# Supplementary material for: How people evaluate anti-corona measures II—Lessons for future crises from a longitudinal study and qualitative analysis
Source: Front Psychol. 2026 Aug 28;17:1806942. doi: 10.3389/fpsyg.2026.1806942 (PMC13561983; doi:10.3389/fpsyg.2026.1806942)
Supplement: Supplementary file 1 [file Data_Sheet_1.pdf]

Supplementary Materials

Table S1: p-values of the t-test comparing empirical value, normed value und total normed value between Study 1 and Study 2 (own representation).

Construct	Empirical value	Normed Value	Total normed value
Attitude	1.66E-08	0.4382	0.4382
Subjective norm	0.3087	0.01101	0.01101
Perceived behavioural control	1.55E-13	6.85E-05	6.85E-05
Close family (attitude)	5.49E-06	0.006297	0.01183
Wider family and friends (attitude)	3.51E-07	0.7156	0.6653
Colleagues at work (attitude)	4.13E-07	0.01464	0.1508
Society in general (attitude)	2.48E-08	0.788	0.9091
Close family (subjective norm)	0.4008	0.02964	0.0004009
Wider family and friends (subjective norm)	0.02531	0.7011	0.08207
Colleagues at work (subjective norm)	0.05424	0.5636	0.01486
Society in general (subjective norm)	0.001901	0.06343	0.6322
Close family (perceived behavioural control)	0.0001536	0.3611	0.1056
Wider family and friends (perceived behavioural control)	5.35E-05	0.5121	0.003988
Colleagues at work (perceived behavioural control)	9.94E-05	0.8044	0.008462
Society in general (perceived behavioural control)	3.05E-05	0.2752	0.002957

Table S2: p-values of the t-test comparing the calculated qualities of the anti-Corna measures and their subordinated constructs – attitude, subjective norm and perceived behavioural control between Study 1 and Study 2 (own representation).

Construct	p-value
Overall quality (restrictions)	< 2.2e-16
Attitude (restrictions)	< 2.2e-16
Subjective norm (restrictions)	< 2.2e-16
Perceived behavioural control (restrictions)	< 2.2e-16
Overall quality (hygiene)	9.36E-11
Attitude (hygiene)	2.80E-05
Subjective norm (hygiene)	1.01E-07
Perceived behavioural control (hygiene)	6.13E-15
Overall quality (mental health)	< 2.2e-16
Attitude (mental health)	< 2.2e-16
Subjective norm (mental health)	< 2.2e-16
Perceived behavioural control (mental health)	< 2.2e-16

Table S3: p-values of the t-test comparing the empirical qualities of the social spheres within the attitude, subjective norm and perceived behavioural control for restrictions on outdoor activities, tips for hygiene and tips for mental health between Study 1 and Study 2 (own representation).

Construct	Restrictions on outdoor activities	Tips for hygiene	Tips for mental health
Attitude: close family	3.44E-16	0.0001585	< 2.2e-16
Attitude: wider family and friends	< 2.2e-16	3.79E-07	< 2.2e-16
Attitude: colleagues at work	4.43E-11	0.0005756	< 2.2e-16
Attitude: society in general	6.96E-13	0.007394	< 2.2e-16
Subjective norm: close family	< 2.2e-16	6.82E-07	< 2.2e-16
Subjective norm: wider family and friends	< 2.2e-16	2.12E-09	< 2.2e-16
Subjective norm: colleagues at work	< 2.2e-16	0.0004838	< 2.2e-16
Subjective norm: society in general	< 2.2e-16	3.40E-11	< 2.2e-16
Percieved behavioural control: close family	< 2.2e-16	3.06E-13	< 2.2e-16
Percieved behavioural control: wider family and friends	< 2.2e-16	3.84E-16	< 2.2e-16
Percieved behavioural control: colleagues at work	0.01545	0.0001007	< 2.2e-16
Percieved behavioural control: society in general	8.89E-05	0.01025	< 2.2e-16

Table S4: Participants' original (German) statements representing the reasons for positively evaluating and criticising the restrictions on outdoor activities (own representation).

Positive and Critical evaluation	Main category	Sub category	Restrictions on outdoor activities
Positive evaluation	Positive evaluation of measures in general	Measure is effective and/or makes sense in general	Wir müssen die aktuelle Situation unter Kontrolle bekommen und daher drastische Maßnahmen unterstützen
		Measure is effective because of case numbers, studies, experts and/or official approval	Die sinkenden Infektionszahlen zeigen ja dass dies nötig ist
		Measure is the best known solution	Es gibt nach meiner Meinung keine andere Möglichkeit die Zahlen zu reduzieren
	Positive evaluation of measures with a goal-orientation	Measure serves the individual protection from the virus	
		Measure helps to protect the society, others and/or risk groups	Ich finde es richtig, da sonst das Gesundheitssystem überlastet ist.
		Measure is a mean to quickly return to a normal life	Ich denke durch die Maßnahmen steigt die Chance schnell wieder zur normalität zurückkehren zu können.
	Positive evaluation of measures with a focus on execution	Measure is well explained and/or easy to understand	
		Measure is practicable and/or not to restrictive in one's daily life	
		Measure is good in general and not only during the pandemic	
		Measure is necessary because appealing to reason does/did not work	Dadurch, dass es mit Vernunftappellen nicht funktioniert hat, müssen solche Beschränkungen sein
Criticism	Criticism of measures in general	Measure is not effective and/or does not make sense in general	Meiner Meinung eher mangelhaft, der Erfolg bleibt aus
		Measure is not effective because of numbers and/or studies	Keine erwiesene Reduktion der Infektionszahlen
		Measure is not sufficiently researched, tested and/or thought trough	Kein wissenschaftlicher Ansatz, sondern Handeln aus Angst. Es fehlt die Innovation und das langfristige vorrausdeneken.
	Criticism of the extend of measures	Measure should be strengthened/enhanced and/or taken earlier	Maßnahmen sollten härter sein
		Measure is too much, too strict and/or too long	Ich finde die Ausgangsbeschränkungen für übertrieben und sehr einschränkend
		There should have been one hard lockdown and then the society should have been opened again	Lieber kurz und knackig statt lang anhaltend und mild
	Criticism of specific rules	Specific rules of a measure are not effective / do not make sense	ist nach 21 Uhr Corona auf einmal noch aktiver? ich glaube kaum!
		Rules contradict themselves and/or are inconsistent	Die Ausgangsbeschränkungen sind an sich widersprüchlich und ergeben somit oftmals keinen Sinn.
		Rules should be regionalised depending on case numbers	Die Maßnahmen müssten sich unterscheiden in den Bundesländern, oder zumindest nur dort zu Einschränkungen kommen wo es extrem viele Infektionen gibt.
		Rules should be consistent and uniform all over the country	Umsetzung sollte für alle Länder gleich gelten.
	Criticism of measures regarding their execution	Knowledge/transparency of measure is low	Viele Beschränkungen sind nicht transparent und nachvollziehbar.
		Rules are difficult to accept/follow and/or are tiering/exhausting oder time	Manche Maßnahmen sind schwierig umzusetzen.
		Others do not sufficiently obey the rules and/or rules should be enforced more strictly	Teilweise wäre es mir lieber wenn die Ausgangsbeschränkungen noch härte kontrolliert und sanktioniert würden, damit mehr Sicherheit gewährleistet ist.
		The vaccination process is poorly organised and/or executed	
	Criticism of measures regarding side-effects	Measure affects quality of life and/or menatl health	Aufgrund dieser Beschränkungen hat sich meine Psyche stark verschlechtert, auch in meinem Bekanntenkreis.
		Measure is a restriction of freedom	Hier wird die Freiheit zu sehr beschnitten.
		Measure causes economic damage	Momentan sind das zu harte Maßnahmen dadurch gehen viele Insolvenz
		Measure may cause unkown (long-term) side-effects	
	Criticism of the indivituality and specificity of measures	A general approach does not work and/or measure should be individualised	
		Measure is too superficial, not specific enough and/or no help for severely affected people	
		Measure cannot compensate the social situation	

Table S5: Participants' original (German) statements representing the reasons for positively evaluating and criticising the tips for hygiene (own representation).

Positive and Critical evaluation	Main category	Sub category	Tips for hygiene
Positive evaluation	Positive evaluation of measures in general	Measure is effective and/or makes sense in general	Allgemein sehr gut
		Measure is effective because of case numbers, studies, experts and/or official approval	Die Tipps sind von verschiedenen Forscher belegt und bestätigt.
		Measure is the best known solution	Es gibt keine Alternative
	Positive evaluation of measures with a goal-orientation	Measure serves the individual protection from the virus	
		Measure helps to protect the society, others and/or risk groups	Die Tipps sind super wichtig, damit man auch andere vor das Virus schützt.
		Measure is a mean to quickly return to a normal life	
	Positive evaluation of measures with a focus on execution	Measure is well explained and/or easy to understand	Ausreichen dargestellt und sinnvoll und nachvollziehbar.
		Measure is practicable and/or not to restrictive in one's daily life	Simple und leicht umsetzbar
		Measure is good in general and not only during the pandemic	Gute Tipps, sollten auch in Zukunft beibehalten werden.
		Measure is necessary because appealing to reason does/did not work	
Criticism	Criticism of measures in general	Measure is not effective and/or does not make sense in general	Im Privaten wird es nicht umgesetzt, in der Öffentlichkeit hatte man vorher meistens eh schon abstand zu Menschen.
		Measure is not effective because of numbers and/or studies	
		Measure is not sufficiently researched, tested and/or thought trough	
	Criticism of the extend of measures	Measure should be strengthened/enhanced and/or taken earlier	Könnte mehr sein. Zu wenige Maßnahmen auch in der Öffentlichkeit
		Measure is too much, too strict and/or too long	
		There should have been one hard lockdown and then the society should have been opened again	
	Criticism of specific rules	Specific rules of a measure are not effective / do not make sense	teilweise unsiinnig
		Rules contradict themselves and/or are inconsistent	Die Aussagen widersprechen sich teilweise.
		Rules should be regionalised depending on case numbers	
		Rules should be consistent and uniform all over the country	
	Criticism of measures regarding their execution	Knowledge/transparency of measure is low	bessere und mehr Informationen zur Hygiene wären sinnvoll.
		Rules are difficult to accept/follow and/or are tiering/exhausting oder time	Umsetzbarkeit nicht immer machbar.<br>z.B. im Handwerk
		Others do not sufficiently obey the rules and/or rules should be enforced more strictly	Wenn sich jede*r daran halten würde, würden die Zahlen sicherlich sinken.
		The vaccination process is poorly organised and/or executed	
	Criticism of measures regarding side-effects	Measure affects quality of life and/or menatl health	Sie lösen bei Kindern Stress und teilweise Angst aus.
		Measure is a restriction of freedom	
		Measure causes economic damage	
		Measure may cause unkown (long-term) side-effects	
	Criticism of the indivituality and specificity of measures	A general approach does not work and/or measure should be individualised	
		Measure is too superficial, not specific enough and/or no help for severely affected people	
		Measure cannot compensate the social situation	

Table S6: Participants' original (German) statements representing the reasons for positively evaluating and criticising the tips for mental health (own representation).

Positive and Critical evaluation	Main category	Sub category	Tips for mental health
Positive evaluation	Positive evaluation of measures in general	Measure is effective and/or makes sense in general	Die Tipps sind gut.
		Measure is effective because of case numbers, studies, experts and/or official approval	
		Measure is the best known solution	
	Positive evaluation of measures with a goal-orientation	Measure serves the individual protection from the virus	
		Measure helps to protect the society, others and/or risk groups	
		Measure is a mean to quickly return to a normal life	
	Positive evaluation of measures with a focus on execution	Measure is well explained and/or easy to understand	
		Measure is practicable and/or not to restrictive in one's daily life	können Umgesetzt werden mit Hilfe
		Measure is good in general and not only during the pandemic	Gute Tipps. Universell. auch außerhalb der Pandemie nützlich
		Measure is necessary because appealing to reason does/did not work	
Criticism	Criticism of measures in general	Measure is not effective and/or does not make sense in general	Ich persönlich finde die Tipps nicht so hilfreich.
		Measure is not effective because of numbers and/or studies	
		Measure is not sufficiently researched, tested and/or thought trough	
	Criticism of the extend of measures	Measure should be strengthened/enhanced and/or taken earlier	Selbstmordrate steigt expotentiell an - hier muss einiges mehr getan werden!
		Measure is too much, too strict and/or too long	
		There should have been one hard lockdown and then the society should have been opened again	
	Criticism of specific rules	Specific rules of a measure are not effective / do not make sense	
		Rules contradict themselves and/or are inconsistent	
		Rules should be regionalised depending on case numbers	
		Rules should be consistent and uniform all over the country	
	Criticism of measures regarding their execution	Knowledge/transparency of measure is low	Habe ich bisher noch nicht viel von mitbekommen
		Rules are difficult to accept/follow and/or are tiering/exhausting oder time	Schwierig umzusetzen weil man es oft im Alltag vergisst.
		Others do not sufficiently obey the rules and/or rules should be enforced more strictly	
		The vaccination process is poorly organised and/or executed	
	Criticism of measures regarding side-effects	Measure affects quality of life and/or menatl health	
		Measure is a restriction of freedom	
		Measure causes economic damage	
		Measure may cause unkown (long-term) side-effects	
	Criticism of the indivituality and specificity of measures	A general approach does not work and/or measure should be individualised	zu allgemein. JEder braucht seine eigenen Tipps
		Measure is too superficial, not specific enough and/or no help for severely affected people	Netter Ansatz, aber wenn jemand wirklich unter einer Angsstörung o.ä. leidet
		Measure cannot compensate the social situation	Diese Tipps helfen meiner seelischen Gesundheit nicht. Was einzig hilft, sind soziale Kontakte.

Table S7: Participants' original (German) statements representing the reasons for positively evaluating and criticising the vaccination (own representation).

Positive and Critical evaluation	Main category	Sub category	Vaccination
Positive evaluation	Positive evaluation of measures in general	Measure is effective and/or makes sense in general	Ich bin davon überzeugt
		Measure is effective because of case numbers, studies, experts and/or official approval	Ich vertraue den Fachleuten und dem Zulassungsprozess.
		Measure is the best known solution	Es ist die einzige Lösung für ein Ende der Pandemie
	Positive evaluation of measures with a goal-orientation	Measure serves the individual protection from the virus	Ich halte sehr viel davon, denn mit einer Impfung sind wir auch langfistig vor Corona geschützt
		Measure helps to protect the society, others and/or risk groups	Sinnvoll um besonders die gefährdeten Menschen zu schützen
		Measure is a mean to quickly return to a normal life	Sie wird einen normaleren Alltag zulassen
	Positive evaluation of measures with a focus on execution	Measure is well explained and/or easy to understand	
		Measure is practicable and/or not to restrictive in one's daily life	
		Measure is good in general and not only during the pandemic	
		Measure is necessary because appealing to reason does/did not work	
Criticism	Criticism of measures in general	Measure is not effective and/or does not make sense in general	jedem das seine. ich halte nicht so viel von
		Measure is not effective because of numbers and/or studies	
		Measure is not sufficiently researched, tested and/or thought trough	Nicht ausreichend erforscht
	Criticism of the extend of measures	Measure should be strengthened/enhanced and/or taken earlier	
		Measure is too much, too strict and/or too long	
		There should have been one hard lockdown and then the society should have been opened again	
	Criticism of specific rules	Specific rules of a measure are not effective / do not make sense	
		Rules contradict themselves and/or are inconsistent	
		Rules should be regionalised depending on case numbers	
		Rules should be consistent and uniform all over the country	
	Criticism of measures regarding their execution	Knowledge/transparency of measure is low	Dazu sind zu wenige Informationen bekannt für die Bürger
		Rules are difficult to accept/follow and/or are tiering/exhausting oder time	
		Others do not sufficiently obey the rules and/or rules should be enforced more strictly	
		The vaccination process is poorly organised and/or executed	Der Prozess der Impfung in Deutschland ist nicht organisiert genug.
	Criticism of measures regarding side-effects	Measure affects quality of life and/or menatl health	
		Measure is a restriction of freedom	
		Measure causes economic damage	
		Measure may cause unkown (long-term) side-effects	Es ist zu wenig über die Nachwirkungen bekannt.
	Criticism of the indivituality and specificity of measures	A general approach does not work and/or measure should be individualised	
		Measure is too superficial, not specific enough and/or no help for severely affected people	
		Measure cannot compensate the social situation	
